# Identification of a PRDM1-regulated T cell network to regulate atherosclerotic plaque inflammation

**DOI:** 10.1186/s13073-025-01541-6

**Published:** 2025-10-02

**Authors:** Han Jin, Sanne L. Maas, Yuchi Zou, Chang Lu, Baixue Yu, Rosanna Huchzermeier, Samantha Nadeau, Jessica Dos Santos, Marion Gijbels, Barend M. E. Mees, Evgueni Smirnov, Ljubica Matic, Ulf Hedin, Pasquale Maffia, Claudia Monaco, Judith C. Sluimer, Gislâine A. Martins, Emiel P. C. van der Vorst, Erik A. L. Biessen

**Affiliations:** 1 Central Laboratory, Tianjin Medical University General Hospital, Tianjin, China; 2 Department of Pathology, Cardiovascular Research Institute Maastricht (CARIM), Maastricht UMC+, P. Debyelaan 25, Maastricht, HX 6229 the Netherlands; 3 Department of Internal Medicine I, University Hospital Aachen, RWTH Aachen University, Aachen, Germany; 4 Institute for Molecular Cardiovascular Research (IMCAR), Uniklinik RWTH Aachen, RWTH Aachen University, Aachen, Germany; 5 Aachen-Maastricht Institute for Cardio-Renal Disease (AMICARE), Uniklinik RWTH Aachen, RWTH Aachen University, Aachen, Germany; 6 Departments of Medicine and Biomedical Sciences, David Geffen School of Medicine, Research Division of Immunology Cedars-Sinai Medical Center, University of California, Los Angeles, CA USA; 7 Department of Medical Biochemistry, Amsterdam Cardiovascular Sciences: Atherosclerosis & Ischemic Syndrome; Amsterdam Infection and Immunity: Inflammatory diseases; Amsterdam UMC location University of Amsterdam, Amsterdam, the Netherlands; 8 Department of Vascular Surgery, Maastricht UMC+, Maastricht, the Netherlands; 9 Department of Data Science and Knowledge Engineering, Maastricht University, Maastricht, the Netherlands; 10 Division of Vascular Surgery, Department of Molecular Medicine and Surgery, Karolinska Institutet and Karolinska University Hospital, Karolinska, Sweden; 11 School of Infection & Immunity, College of Medical, Veterinary and Life Sciences, University of Glasgow, Glasgow, UK; 12 Department of Pharmacy, School of Medicine and Surgery, University of Naples Federico II, Naples, Italy; 13 Africa-Europe CoRE in Non-Communicable Diseases & Multimorbidity, African Research Universities Alliance (ARUA), The Guild of European Research-Intensive Universities, Glasgow, UK; 14 Nuffield Department of Orthopaedics, Rheumatology and Musculoskeletal Sciences, Kennedy Institute of Rheumatology, University of Oxford, Oxford, UK; 15 Centre for Cardiovascular Science, University of Edinburgh, Edinburgh, Scotland; 16 Inflammatory Bowel and Immunobiology Research Institute, Cedars-Sinai Medical Center, Los Angeles, CA USA; 17 Institute for Cardiovascular Prevention (IPEK), Ludwig-Maximilians-University Munich, Munich, Germany

**Keywords:** Carotid endarterectomy, T cell, Microarray, Single-cell sequencing, Atherosclerosis, PRDM1

## Abstract

**Background:**

Inflammation is a key driver of atherosclerosis, yet the mechanisms sustaining inflammation in human plaques remain poorly understood. This study uses a network-based approach to identify immune gene programs involved in the transition from low- to high-risk (rupture-prone) human atherosclerotic plaques.

**Methods:**

Expression data from human carotid artery plaques, both stable (low-risk, *n* = 16) and unstable (high-risk, *n* = 27), were analyzed using Weighted Gene Co-expression Network Analysis (WGCNA). Bayesian network inference, operated on the eigengene values from the WGCNA, further extended the WGCNA analysis, and similarity to the signature of T cell subsets was validated in single-cell RNA sequencing data of human plaques, and a loss-of-function study in a mouse model of atherosclerosis. In silico drug repurposing was performed to identify potential therapeutic targets.

**Results:**

Our analysis revealed a distinct gene module with a prominent T cell signature, particularly in unstable plaques. Key regulatory factors, RUNX3, IRF7 and in particular PRDM1, were significantly downregulated in plaque T cells from symptomatic versus asymptomatic patients, indicating a protective role. Additionally, as PRDM1 is downstream of IRF7, we opted for PRDM1 as a key target. T cell-specific *Prdm1* deficiency in Western-type diet fed *Ldlr* knockout mice featured accelerated plaque progression. Finally, as PRDM1 targeting drugs are not yet available, we performed in silico drug repurposing, identifying EGFR inhibitors as promising therapeutic candidates.

**Conclusions:**

This study highlights a PRDM1-regulated T cell network that distinguishes high-risk from low-risk plaques and demonstrates the regulatory role of T cell PRDM1 in controlling atherosclerosis, positioning this pathway as a promising therapeutic target.

**Supplementary Information:**

The online version contains supplementary material available at 10.1186/s13073-025-01541-6.

## Background

Atherosclerosis is characterized by the intimal accumulation of lipoproteins, debris, and immune cells in medium-large sized arteries [1]. Atherosclerosis progression toward a rupture-prone plaque, at risk of causing an ischemic event, is a complex and multifactorial process. Plaque progression involves both innate and adaptive immune responses, alongside angiogenesis, extracellular matrix deposition, erosion, and dysfunction of vascular smooth muscle cells (VSMCs) [2–4] Recent studies have highlighted the potential of immunotherapy in preventing atherosclerosis-related cardiovascular events [5–7], spurring global efforts in both pharmaceutical industry and academia to develop targeted interventions for plaque inflammation. However, most research has focused on single factors involved in plaque inflammation, thereby overlooking the complex inter-gene interactions and the inherent relationships between the underlying biological pathways and disease progression. To truly understand and combat atherosclerosis, a more holistic approach that examines the broader network of relevant pathways is essential.

The use of high-throughput technologies like microarray and RNA sequencing (RNA-seq) has significantly advanced our understanding of key processes driving plaque progression and destabilization [8, 9]. More recently, single-cell RNA-seq (scRNA-seq) has enabled the analysis of gene expression in specific cell populations within diseased tissue, including mouse and human atherosclerotic plaque. These studies uncovered a remarkable heterogeneity of key immune populations and identified subsets of VSMCs, macrophages, and T cells linked to disease progression [10–12]. Additionally, scRNA-seq has facilitated the discovery of critical crosstalk between different cell populations that contributes to plaque development [13, 14].

Although scRNA-seq studies have been invaluable in identifying potential drug targets and biomarkers, they often fail to capture the full complexity of atherosclerotic plaque tissue. To overcome this limitation, we applied an unbiased computational approach to investigate the changes occurring across the entire plaque as it transitions from a stable (low-risk) to an unstable plaque (high-risk, rupture-prone) state. Using global gene expression profiles from symptomatic carotid artery lesions in the Maastricht Human Plaque Study (MaasHPS) cohort (*n* = 43, with 16 stable and 27 unstable plaque samples) [15–18], we performed gene co-expression and regulatory network analysis. This approach identified a critical T cell-associated network that plays a key role in driving this phenotypic transition. Additionally, we validated the functional significance of a key regulatory signal within this network through a loss-of-function study in a mouse model of atherosclerosis, further supporting its role in plaque destabilization.

## Methods

A list of materials can be found in Additional file 1: Materials.

### Datasets

The microarray data used in this study is derived from analyses of a previously published dataset by our laboratory, namely the MaasHPS [18] (Table 1). Detailed descriptions of sample collection, preparation, and microarray data generation have been reported in the following publications. [15–17] For the sake of clarity and completeness, key aspects of the sample handling and microarray procedures are summarized in the Methods sections below (sections “ Human sample collection,” “ Morphometry,” “ Sample preparation,” “ Microarray data collection,” “ Microarray data pre-processing”). Information on all datasets used in this study can be found in the table below (Table 1).

**Table 1:** Description of datasets used in this study

Human plaque dataset	Sample size	Study design	Analyses	GEO accession	References
MaasHPS microarray	43	Stable (*n* = 16) Unstable (*n* = 27)	Differential expression; WGCNA; Bayesian network; Regulatory network	GSE163154	[15–18]
BiKE microarray	137	Atherosclerotic tissue (*n* = 127); Non-atherosclerotic artery tissue (*n* = 10)	Differential expression	GSE21545	[8, 19–21]
Decoding the transcriptome of calcified atherosclerotic plaque at single-cell resolution	6	Atherosclerotic core (*n* = 3); Proximal adjacent portion (*n* = 3)	Differential expression	GSE159677	[22, 23]
Single-cell immune landscape of human atherosclerotic plaques	6	Symptomatic (*n* = 2) Asymptomatic (*n* = 4)	Differential expression	GSE224273	[13, 24, 25]

### Human sample collection

**Fig. 1:**
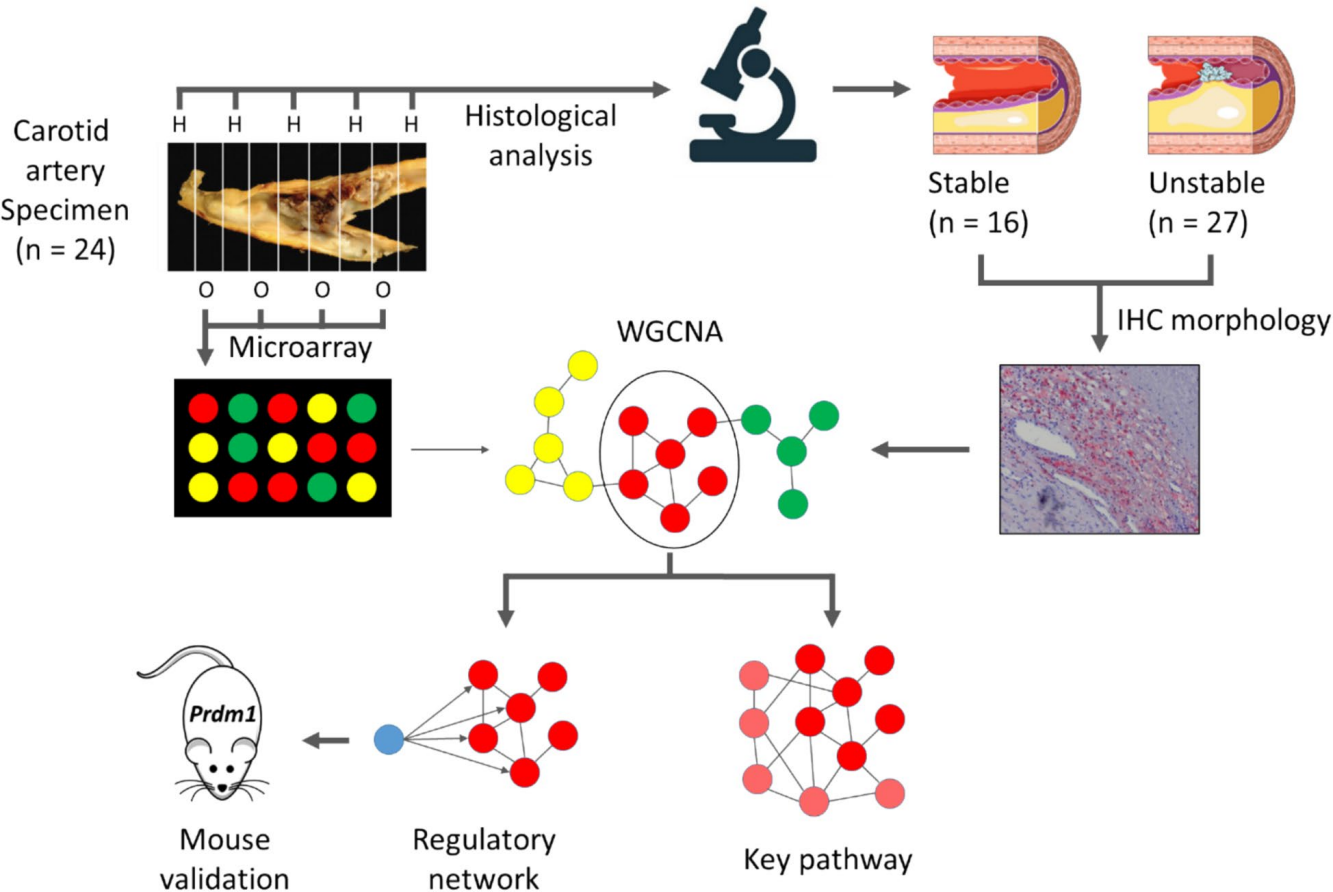
Schematic workflow. The carotid artery specimen were sliced into 5 mm thick parallel sections, snap-frozen in liquid nitrogen, and stored for future use. Every second section was used for histology, stained with Hematoxylin and Eosin, and classified based on the absence/presence of IPH and classification criteria adapted from the American Heart Association (AHA) scheme [26] to determine progression stage. This classification yielded 16 stable (low-risk) and 27 unstable (high-risk) plaque segments, which were subsequently used for microarray analysis. WGCNA was conducted on the microarray gene expression data from the carotid endarterectomy plaques. Co-expression clusters were identified through network-based hierarchical clustering, and disease-associated clusters were pinpointed by integrating WGCNA results with plaque traits from IHC. These selected clusters were then further examined through gene set enrichment, overrepresentation, correlation, and regulatory network analysis. One key regulator identified in the regulatory network analysis, PRDM1, was investigated in an atherosclerotic mouse model, specifically examining the effect of T cell-specific *Prdm1 *deficiency on atherosclerosis development. AHA - American Heart Association, IHC - Immunohistochemistry, PRDM1 - PR Domain Zinc Finger Protein 1, WGCNA - Weighted Gene Co-expression Network Analysis

Atherosclerotic plaque samples obtained during carotid endarterectomy (CEA) from 24 symptomatic patients (all male, age = 72.84 ± 6.47, mean ± SD; SD, standard deviation) were collected in the Maastricht Pathology Tissue Collection (MPTC). For detailed information on the patient cohort definition, see Additional file 2: Table S1-1. The CEA specimens were cut into parallel transverse segments of 5-mm thickness. Each alternating segment was snap-frozen in liquid nitrogen and stored at − 80 °C, the flanking segments were fixed in formalin and processed for histological evaluation (Fig. 1).

Plaque tissue segments were stratified into low-risk and high-risk groups. High-risk plaques are more prone to rupture or erosion, events that can trigger acute cardiovascular complications such as myocardial infarction or stroke. In this study, plaques are classified as stable (SP) or unstable plaques (UP) based on histopathological plaque staging by two expert pathologists, according to the well-established Virmani classification scheme adapted from the American Heart Association [26]. Plaques were classified as stable plaques if they exhibited features of pathological intimal thickening, early or late fibroatheromas, or fibrocalcified plaques, and as unstable plaques if they showed evidence of intraplaque hemorrhage (IPH), ruptured plaques, and acute occlusions. The presence of IPH was assessed by computer-aided quantitative measurement of extravascular erythrocyte deposits in the hematoxylin–eosin-stained (H&E) section, flanking the omics section. Stable and unstable plaque segments were collected from 21 out of 24 patients, from the remaining three patients two distinct unstable plaque segments were collected, resulting in a total of 48 segments (21 stable plaques and 27 unstable plaques). Five segments were excluded from the analysis based on inconsistent plaque score assessment by the observers (*n* = 1), sub-threshold RNA quality (*n* = 3), or microarray quality control (*n* = 1), resulting in a total of 43 segments (16 stable plaques and 27 unstable plaques), which were subjected to transcriptomic profiling. We refer to the sample preparation below. Further details about the characteristics of the patients of the MaasHPS cohort [18](including sex, age, use of medications, lipid profiles, and other cardiovascular risk factors, Additional file 2: Table S1-1) and the inclusion/exclusion criteria of this cohort have been reported in our previous publications. [15–17]

### Morphometry

Morphometric analysis was performed on CEA sections that were stained with H&E. Slides were photographed at × 12.5 magnification and examined digitally using Leica Q500MC software. Total tissue, media, fibrous cap, necrotic core, hemorrhage, and luminal thrombus area were measured. Plaque size was calculated by subtracting the medial and luminal thrombus area from that of the total carotid tissue. One stable segement was excluded due to a inconsistent plaque score assessment by the observers (*n = 1*). In addition, staining’s were performed on CEA sections to measure the relative area or cell counts of the following histological features: CD3^+^ T cell density: CD3^+^ cells/plaque area (n/mm^2^); CD31^+^ endothelial cell (EC) density: CD31^+^ lumen-lining cells/plaque area (n/mm^2^); CD31^+^ microvessel density: CD31^+^-lined structures with a lumen/plaque area (n/mm^2^); D2-40^+^ lymphangiogenesis density: D2-40^+^ microvessels/plaque area (n/mm^2^); CD68^+^ macrophage density: CD68^+^ cells/plaque area (n/mm2); iNOS^+^ (M1)-like macrophage fraction iNOS^+^ area/CD68^+^ area (n/mm^2^); Arg1^+^ (M2)-like macrophage fraction: Arg1^+^ area/CD68^+^ area (n/mm^2^); collagen content: % Sirius red of total plaque area; alpha-smooth muscle actin (αSMA^+^) positive smooth muscle cell (SMC) density: αSMA^+^ cells/plaque area (n/mm^2^); and calcification: % Alizarin red area of total plaque area.

### Sample preparation

Snap-frozen omics segments were pulverized and 5 to 20 mg of material was subjected to microarray analysis. RNA was isolated by guanidium thiocyanate extraction, followed by further purification using the Nucleospin RNA II kit (Macherey–Nagel GmbH & Co. KG). RNA concentrations were measured using a Nanodrop ND-1000 spectrophotometer (Nanodrop Technologies, Wilmington, DE, U.S.A). RNA quality and integrity were determined with the Agilent 2100 Bioanalyzer (Agilent Technologies, Inc., Santa Clara, CA, U.S.A.). Four stable plaque segments were excluded because of the low RNA quality (RNA Integrity Number (RIN) < 6.0 and A260/280 ratio lower than 1.8; *n* = 3) or failed omics experiment due to technical limitations (*n* = 1). Details have been described in our previous publication [15]. Finally, a total of 43 (16 stable plaques and 27 unstable plaques) samples were used for transcriptional profiling.

### Microarray data collection

Biotinylated cRNA was prepared using the Illumina TotalPrep RNA Amplification Kit (Ambion, Inc., Austin, TX, U.S.A.) according to the manufacturer’s specifications starting with 100 ng total RNA. Per sample 750 ng of cRNA was used for hybridization. Hybridization and washing were performed according to the Illumina standard assay procedure. Scanning was performed on the Illumina BeadStation 500 (Illumina, Inc., San Diego, CA, U.S.A.), while image analysis and extraction of raw expression data were done using Illumina Beadstudio v3 Gene Expression software with default settings, no background subtraction, and no normalization. Transcripts were measured by Illumina HumanRef-8 v2.0 expression BeadChip.

### Microarray data pre-processing

A total of 22,184 human transcripts and variants, as defined by RefSeqs (NCBI) sequences, were analyzed using the R Bioconductor lumi [27] package (v2.38.0). First, we performed a variance stabilizing transformation. Then, the robust spline normalization (RSN) algorithm was applied to normalize the data. If multiple transcripts were detected for a single gene, the highest expressed transcript was used for further analysis, resulting in 18,189 unique genes. As low-variance genes and noisy gene expression reduce the effectiveness of Subsequent clustering analysis, genes below the first quartile for mean absolute deviation scores were discarded. Finally, a total of 13,641 genes could be assigned according to the HUGO Gene Nomenclature Committee (HGNC) nomenclature [28] and their expression data were used for downstream analyses.

### Gene co-expression analysis

Weighted Gene Co-expression Network Analysis (WGCNA) [29] was carried out separately on the pre-processed gene expression data for the subcohorts of stable plaques (*n* = 16) and unstable plaques (*n* = 27). Both networks achieved a scale-free topological structure, with scale-free fitting indices of 0.84 (stable plaques) and 0.81 (unstable plaques) at a soft-thresholding power of six. The adjacency matrix of gene co-expression similarity was calculated for both networks, based on Pearson’s correlation coefficient between gene pairs followed by soft-thresholding power transformation. Then, the adjacency matrix was transformed into a topological overlap matrix (TOM) representing dissimilarity between gene pairs. Hierarchical clustering was performed on both TOMs for both plaque phenotypes with the MEDissThres set as 0.25 to merge similar clusters, resulting in 25 and 38 co-expression clusters after WGCNA on stable and unstable plaques, respectively. The co-expression clusters identified from the WGCNA were named SP-1 to SP-25 for stable plaques and UP-1 to UP-38 for unstable plaque WGCNA.

For the unstable plaque WGCNA clusters, we calculated the eigengene as the first principal component of the cluster and measured the cluster’s overall gene expression. Afterward, these eigengenes were used in the following correlation analysis, the Bayesian network construction, and the visualization of the hierarchical tree dendrogram of unstable plaque WGCNA clusters.

### Differential expression analysis

As described in our previous study [15], differential gene expression analysis of microarray data between stable and unstable plaques was performed by the limma R package (v3.42.2) [30]. The Benjamini–Hochberg procedure was performed to adjust the *p*-values for multiple testing and control the false discovery rate.

### Gene set overrepresentation and enrichment analysis

Gene set overrepresentation analysis (GSOA) was performed for relevant co-expression gene clusters using the R package clusterProfiler (v3.12.0) [31]. Three categories of gene ontology (GO) knowledgebase were queried: biological process, molecular function, and cellular component. Benjamini–Hochberg adjusted *p*-values were calculated, and the cut-off for significance was set to 0.05. Gene ratios were determined representing the percentage of genes associated with the given GO term to the total number of genes in the WGCNA cluster. GO semantic similarity between different WGCNA clusters was measured by the R package GOSemSim (v2.20.0) [32] based on the significant GO biological process terms of each cluster. In addition, for each co-expression cluster, we defined cluster enrichment score (CES) as the absolute log-transformed (base 10) adjusted *p*-value of the most significant GO term associated with this cluster. The overlapping genes between two clusters were evaluated by the Jaccard Index (the intersection over the union set) and by hypergeometric test. GSOA results can be found in Additional file 3: Table S2.

To assess if a co-expression cluster was significantly upregulated in unstable plaque condition, gene set enrichment analysis (GSEA) was performed for unstable plaque WGCNA co-expression clusters against the 13,641 pre-processed genes sorted by log2 fold change, using the R package clusterProfiler (v3.12.0) [31]. GSEA results can be found in Additional file 4: Table S3.

### Bayesian network analysis using the unstable plaque WGCNA eigengenes

Bayesian network inference was performed on the eigengenes of unstable plaque WGCNA, to infer causal relationships between the biological processes derived from GSOA on unstable plaque WGCNA clusters. For clarity, only clusters with CES > 3 (i.e., adjusted *p*-value < 0.001) were selected for Bayesian network construction. The Bayesian network for unstable plaque WGCNA clusters was created using previously described parameter settings [33, 34], based on the hill-climbing strategy in a bootstrapping manner (bootstrap replicates = 1000, aggregate threshold = 0.85), and weaker edges (strength < 0.7) were removed. Finally, a directed acyclic Bayesian network was constructed, which consisted of 15 clusters (nodes) and 28 edges representing the inter-cluster relationships. Separate clusters were tagged to reflect the dominant overrepresented GO according to the corresponding WGCNA. The Bayesian network analysis was performed by the R package bnlearn (v4.5) [35].

### Gene regulatory network reconstruction

Gene expression-based regulatory network (GRN) reconstruction was performed by GEne Network Inference with Ensemble of trees (GENIE3), a machine learning tree-based variable selection method [36]; and Algorithm for the Reconstruction of Accurate Cellular Networks (ARACNe), an information-theoretic method [37]. These two data-driven and computation-based methods generate GRN from the expression data. All 43 samples and 13,641 genes were involved in the analysis. Lambert et al. [38] published a catalog of 1639 human transcription factors (TFs), 1021 of these TFs were identified in our dataset and were selected as candidate TFs. GENIE3 was performed by the R package GENIE3 (v1.6.0) [36], with the candidate TFs as regulators, and all 13,641 genes as targets. ARACNe was performed using the R package minet (v3.42.0) [39] on the full, pre-processed dataset. Then, the 1021 candidate TFs were indicated as regulators targeting the whole ARACNe GRN. Overall, both GENIE3 and ARACNe GRNs had the same scales, with 1021 TFs and 13,641 targets. Furthermore, they displayed scale-free fitting indices of 0.96 and 0.90, respectively, suggestive of scale-free network topology. For both GENIE3 and ARACNe GRNs, the top 100 highest ranked TFs targeting the 250 genes of the unstable plaque WGCNA T cell-specific cluster (UP-32) were selected based on the sum of weighted connections from a TF to its targets (node degree). This resulted in the identification of 32 TFs detected by the two GRNs.

Motif-based TF prediction was performed by iRegulon (v1.3) [40], a Cytoscape [41] plugin, where all 250 genes in the UP-32 “T cell” cluster were used as input. The iRegulon is a knowledge-driven method searching for the best motif from the database that can bind the most input gene targets. A broad search including 9713 position weight matrices and 1120 chromatin immunoprecipitation-sequencing tracks was performed. The putative regulatory region was set at 20 kb, centered on transcription start sites. The normalized enrichment score was set to three to filter out less enriched TFs. Finally, 166 TFs were predicted as drivers of the unstable plaque WGCNA T cell-specific cluster (UP-32). Of the 32 TFs identified by GENIE3 and ARACNe, nine overlapped with iRegulon. GSOA was performed on the selected nine TFs to discover associated biological processes.

### Visualization of WGCNA and GRN

Unstable plaque WGCNA network and GRN for the T cell-specific cluster were visualized by Cytoscape (v3.8.0) [41]. Edges between genes were obtained from the power-transformed adjacency matrix. For visualization purposes, we applied a cut-off by removing edges with a weight > 0.3 to down-scale the size of the network, resulting in 5370 gene nodes (from a total of 12,341 gene nodes) presented in the network. This gene set was projected into a two-dimensional space based on the gene expression in the unstable plaque cohort and denotation according to the unstable plaque WGCNA clusters, using t-Distributed Stochastic Neighbor Embedding (t-SNE) provided in PRESTO (v1.1), a Matlab-based stand-alone software tool for omics analysis [42]. The selected nine TFs, regulating 195 of all 250 gene targets within the T cell-specific cluster (UP-32), were visualized as a GRN by Gephi (v0.9.2) [43], with edges presented by integrating TF-targets regulatory relationships, as predicted by iRegulon, and the weights of edges inferred by GENIE3.

### Single-cell RNA sequencing analysis of human carotid plaques

Two scRNA-seq datasets of human carotid plaques (GSE159677 and GSE224273) were analyzed in this study [13, 22–25]. The former one includes three atherosclerotic core samples (AC) and patient-matched proximal adjacent (PA) portions collected from three patients (for both *n = 3*). The latter contains six plaque samples from symptomatic and asymptomatic patients (*n* = 2 for symptomatic; *n* = 4 for asymptomatic).

For both datasets, raw fastq files were downloaded from GEO using sra-tools (v3.1.1) and were subsequently processed using Cell Ranger (v8.0.0) based on the reference genome (version GRCh38-2024-A) downloaded from the 10 × website. One sample in GSE224273 was sequenced by Cellular Indexing of Transcriptomes and Epitopes by Sequencing (CITE-seq), and for simplicity, only the mRNA-derived cDNA sequencing file was processed, without the inclusion of Antibody-Derived Tag/Hashtag Oligonucleotide (ADT/HTO)-derived cDNA.

Both two datasets were analyzed in a similar manner by the R package Seurat (v5.1.0) [44]. Protein-coding and immunoglobulin (IG) and T cell receptor (TR) genes were included for downstream analyses. Cells that expressed genes between 200 and 5000, UMI counts below 20,000, and mitochondrial contamination below 10% were retained. Genes expressed by less than 30 cells in each dataset (on average five cells per sample) were filtered out. Doublets were removed by DoubletFinder (v2.0.4) [45] in a sample-wise manner. After preprocessing, 45,397 and 10,757 cells were retrained in GSE159677 and GSE224273. Data were normalized by the NormalizeData function, with the top 2000 most variable genes selected by FindVariableFeatures. Data were further scaled by the ScaleData function, with the number of genes and mitochondrial contamination regressed out. Principal component analysis was used for dimensionality reduction, followed by patient-level batch correction by Harmony (v1.2.0) [46]. For GSE159677 and GSE224273, 30 and 20 harmony embedding’s were used for uniform manifold approximation and projection (UMAP) visualization and cell clustering based on the Leiden algorithm, with resolution = 0.3 and 0.5, respectively. Additionally, for GSE159677, 17,030 T cells were reclustered for the subset identification based on conventional T cell markers in a similar manner to global analysis. Gene members in the UP-32 T cell cluster from the bulk analysis were examined for their enrichment in scRNA-seq by the AddModuleScore function. Gene differential analysis was done by the FindMarkers function, and *p*-values were corrected by Benjamini–Hochberg procedure.

### BiKE

The relevance of our regulatory hub PR Domain Zinc Finger Protein 1 (PRDM1) for human atherosclerotic cardiovascular disease (ASCVD) was analyzed in the Biobank of Karolinska Endarterectomies (BIKE) cohort of CEA-derived carotid tissue (*n = 127*) [21]. Details of patient inclusion, surgery and enrolment in the biobank, clinical data, sample processing, and large-scale datasets have been previously described [8, 19, 20]. Normal vascular tissues, 9 iliac arteries and 1 aorta, were collected from organ donors with no history of cardiovascular disease (*n = 10*). For detailed information on the patient cohort definition, see Additional file 2: Table S1-2.

### Mouse experiments

Bone marrow cells (1 × 10^6^/mouse) from T cell-specific *Prdm1*^−/−^ (*CD4-*^*Cre*+^*/Prdm1*^*flox/flox*^; CD4 promotor also deletes *Prdm1* in CD8 T cells) and matched wildtype control mice (*Prdm1*^*flox/flox*^) (described in [47] and kindly provided by Dr. Martins) were isolated from the femur and tibia cavities. Subsequently, the cells were administered to low-density lipoprotein receptor knockout (*Ldlr*^−*/*−^*)* recipient mice (mixed gender; irradiated at 8 weeks of age) by lateral tail vein injection 1 day after a lethal dose of whole-body irradiation (2 × 6 Gy). After 5 weeks of recovery, the mice were fed a Western-type diet (WTD) containing 0.25% cholesterol (Special Diet Services 824,171) for 12 weeks. All of the mice were housed under specific pathogen-free conditions in 12 h/12 h light–dark cycles at 21 °C and 50% humidity with ad libitum food and water. Mice were anesthetized (10 mg/kg Xylazin and 100 mg/kg Ketamin in 0.9% NaCl), the blood was collected via puncture of the retro-orbital plexus, and the vasculature was perfused with PBS (Sigma–Aldrich Cat#P7059).

### Histology and immunofluorescent stainings

Atherosclerotic lesion size was assessed in the aortic root and aortic arches. In brief, hearts were embedded in Tissue-Tek O.C.T. compound (Sakura) for cryosectioning of the aortic root. The plaques of the aortic roots were quantified in 5-µm transverse sections stained with H&E. The average plaque size was calculated from three sections. Valves of the aortic root were examined independently and classified as early progressive (pathologic intimal thickening, PIT), advanced with a thick fibrotic cap (TkF), or advanced with a thin fibrotic cap (TnF) as described before (Additional file 5: Fig S1) [48, 49]. The cellular composition of the aortic roots was analyzed. Macrophage content was determined by anti-Macrophage antigen 2 (Mac2) (Cedarline), followed by a 30-min secondary Cy-3-conjugated antibody incubation (Life Technologies). After counter-staining of nuclei with 4',6-diamidino-2-phenylindol (DAPI), the sections were embedded with VectaShield Hard Set Mounting Medium (Vector Laboratories). Aortic roots were analyzed using a Leica DMLB fluorescence microscope with a charge-coupled device (CCD) camera. Collagen was visualized and analyzed in aortic root sections stained with Masson’s Trichrome. These sections were also used for necrotic core size analysis (defined as anucleated area). Aortic arches were isolated (including the main branch points: brachiocephalic artery, left subclavian artery and left common carotid artery) fixed with 4% paraformaldehyde and embedded in paraffin. Lesion size was quantified in 3-µm transverse H&E-stained sections. The average plaque size was calculated from four sections. All analyzed images were performed blinded by using ImageJ software.

### Flow cytometry

Whole blood from T cell-specific *Prdm1*^−/−^ and matched wildtype control mice were collected in ethylenediaminetetraacetic acid (EDTA) buffer tubes. The spleens were isolated, mechanically crushed and passed through a 30-μm cell strainer (Cell-Trics, Partec) using Hank’s Medium (Hanks’ Balanced Salt Solution + 0.3 mmol/l EDTA + 0.1% BSA; Gibco by life technologies) to obtain single-cell suspensions. For further analysis using flow cytometry, the blood as well as the spleen samples was subjected to red-blood-cell lysis. Bone marrow cells were collected by flushing femur cavities with Hank’s Medium. Inguinal lymph nodes were also mechanically crushed, passed through a 30-μm cell strainer (Cell-Trics, Partec) to obtain single-cell suspensions. Subsequently, single-cell suspensions were exposed to fluorescently labeled antibodies and analyzed using a FACS Canto II with the FACSDiva software (BD Biosciences). Cell populations were discriminated by the following: anti-CD45, anti-CD115, anti-Gr1, anti-CD11b, anti-B220, anti-CD3, anti-CD4, and anti-CD8 (All eBioscience, anti-Gr1 Biolegend). Cell populations were gated using the FlowJo analysis program (Treestar): neutrophils (CD45^+^CD115^−^Gr1^high^), classical monocytes (CD45^+^CD11b^+^CD115^+^Gr1^high^), non-classical monocytes (CD45^+^CD11b^+^CD115^+^Gr1^low^), B cells (CD45^+^CD115^−^Gr1^low^B220^+^), T cells (CD45^+^CD115^−^Gr1^low^B220^−^CD3^+^), CD3^+^CD8^+^ T cells (CD45^+^CD115^−^Gr1^low^B220^−^CD3^+^CD8^+^), CD3^+^CD4^+^ T cells (CD45^+^CD115^−^Gr1^low^B220^−^CD3^+^CD4^+^), and CD3^+^CD4^+^CD25^+^ T cells (CD45^+^CD115^−^Gr1^low^B220^−^CD3^+^CD4^+^CD25^+^).

### Plasma lipid levels

Cholesterol and triglyceride levels were analyzed using mouse plasma, isolated from the EDTA-buffered blood. Levels were quantified using enzymatic assays (c.f.a.s. cobas, Roche Diagnostics) according to the manufacturer’s protocol. In short, triglyceride levels were measured in undiluted samples, while cholesterol levels were measured in samples that were diluted 1:5. A standard series of cholesterol and triglycerides was created, after which standards and samples were analyzed in Duplicates. Absorbance was measured at 510 nm with a Tecan Infinite F200 Pro Plate Reader (Tecan Life Sciences Home).

### Drug repurposing on T cell cluster

We performed drug screening and repurposing on the T cell-specific cluster from unstable plaque WGCNA (UP-32). Drug screening was achieved with the use of Library of Integrated Network-Based Cellular Signatures (LINCS) L1000, a database containing genome-wide transcriptional profiles from a broad collection of human cultured cells after perturbation by FDA-approved drugs or small molecules [50]. The highest relevant cell line, i.e., the Jurkat T cells (Clone E6­1, ATCC® TIB­152™), was selected for this study. Eventually, 145 upregulated (log2FC > 0; FC, fold change) and 29 downregulated (log2FC < 0) genes of the T cell-specific cluster, which were also contained in the LINCS database, were selected for drug screening. The connectivity scores between drug-induced gene expression in the LINCS L1000 database and unstable plaque-associated gene expression in the T cell-specific cluster were calculated using the weighted connectivity score as described in the original paper [50] and were further normalized in the range of [−1, 1].

### Statistical analysis

Cluster-trait, gene-trait, and gene-cluster correlations were measured by Pearson’s correlation coefficients with *p*-values. Statistical analyses of human and omics data were performed in R (v4.4.0). Statistical analyses of the murine data were performed using the Prism software (GraphPad Software version 5 or higher, San Diego, CA, USA). Normality was tested and differences between two groups were evaluated for statistical significance with an unpaired Student *t* test with Welch correction (parametric data) or with the Mann–Whitney test (non-parametric data). Data are presented as mean ± SEM. Significance level is denoted by *p-value < 0.05, **p-value < 0.01, ***p-value < 0.001, ****p-value < 0.0001.

### Study approval

The collection of CEA specimens was in line with the Dutch Code for Proper Secondary use of Human Tissue (https://www.federa.org) and approved by the local Medical Ethical Committee (protocol number 16–4-181). No prior informed consent was directly obtained from the patients, but an opt-out arrangement was in place and hence tissues were not used in case of objection, which is in line with the guidelines provided by the Dutch Code for Proper Secondary use of Human Tissue. All mouse experiments were approved by the regulatory authority of Maastricht University Medical Centre and local German regulatory authorities (Landesamt für Natur, Umwelt und Verbraucherschutz Nordrhein-Westfalen. Furthermore experiments were performed in compliance with the guidelines described in Directive 2010/63/EU of the European Parliament and the German animal protection law.

## Results

### Identification of key co-expression clusters in unstable plaques

This study focused on analyzing unstable plaques due to their strong association with an increased risk of cardiovascular events. WGCNA was applied to microarray gene expression profiles from human carotid endarterectomy (CEA) plaques, resulting in the construction of an unstable plaque-specific co-expression network (for research strategy see Fig. 1).

**Fig. 2:**
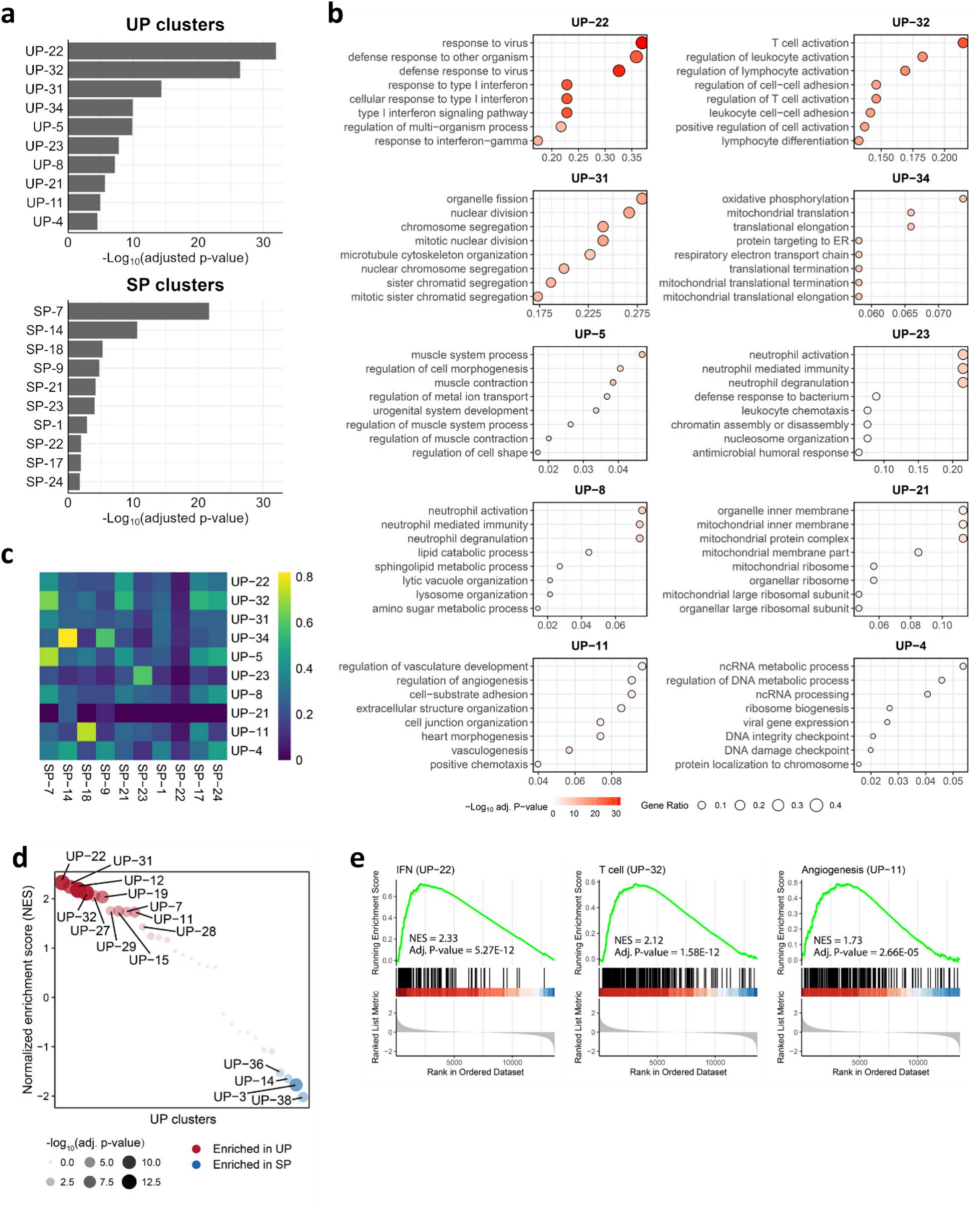
WGCNA of unstable and stable plaques identifies key gene co-expression clusters. **a** Bar plots display the top 10 co-expression clusters ranked by clustering enrichment scores derived from GSOA of WGCNA clusters based on unstable plaques (UP) or stable plaques (SP). **b** Dot plots illustrate the significance levels of the top-ranked overrepresented GO terms for each of the top 10 UP co-expression clusters. **c** GO-based semantic similarity between the top-10 UP clusters and the top-10 SP clusters in **a**. **d** Dotplot summarizes the GSEA results of UP clusters. Significantly enriched clusters (adjusted *p*-value < 0.05) are labeled. **e** GSEA results for the three significant UP clusters (UP-11, UP-22, and UP-32) are presented. Adj – adjusted, IFN – interferon, IPH – intraplaque hemorrhage, GO - Gene Ontology, GSEA - Gene Set Enrichment Analysis, GSOA - Gene Set Overlap Analysis , SP – stable plaque, UP – unstable plaque, WGCNA - Weighted Gene Co-expression Network Analysis

A total of thirty-eight co-expression clusters (UP-1 to UP-38) were identified in the unstable plaques (Additional file 5: Fig S2a). To pinpoint the most biologically relevant clusters within the global network, the top ten co-expression clusters with the highest significance (CES, defined as the absolute log10-transformed adjusted *p*-value of the most significant GO term) were selected through GO-based GSOA (Fig. 2a). The specific enriched GO terms of these top clusters are detailed in Fig. 2b and Additional file 3: Table S2. Among the highest-ranked clusters, UP-22 and UP-32 (labeled “interferon/IFN” and “T cell”, respectively, to reflect their primary enriched GO terms) were strongly linked to immune processes, indicating inflammation is a key feature of unstable plaques. Other high-ranking clusters were associated with processes such as “cell division and proliferation” (UP-4 and UP-31), “mitochondrial activity” (UP-21 and UP-34), “muscle and myocyte” (UP-5), “neutrophil” (UP-8 and UP-23), and “angiogenesis” (UP-11), all representing key biological processes in unstable plaque formation.

To assess whether the network modules were also operational in stable plaques, we constructed a co-expression network for these plaques as well, revealing 25 clusters (SP-1 to SP-25) (Additional file 5: Fig S2b). The top 10 biologically significant clusters are shown in Fig. 2a and Additional file 5: Fig S3a. We then performed a GO-based semantic similarity analysis [32], using enriched GO terms from these clusters to compare the functional correspondence between top-ranked unstable and stable clusters. This analysis revealed four high-ranking unstable plaque clusters (i.e., UP-32 “T cell”; UP-34 “mitochondrial activity”; UP-5 “muscle and myocyte”; and UP-11 “angiogenesis”) that were seen to functionally mirror stable plaque modules (i.e., SP-7, SP-14, SP-7, and SP-18, respectively) as shown by Jaccard similarity, where the gene members were also significantly overlapping (Fig. 2c,Additional file 5: Fig S3b-c and Additional file 6: Table S4). This suggests that these biological processes are already active in the stable plaque stage. By contrast, UP-22 (“IFN”) did not functionally associate with any of the stable plaque clusters (Fig. 2c), suggesting that these processes mainly are relevant in the high-risk, unstable plaque stage.

Subsequent GSEA was performed using gene expression changes in unstable plaques relative to stable plaques to assess whether genes from a cluster were significantly upregulated in the unstable condition. This analysis revealed a significant overall upregulation of gene members of three of the four unstable plaque clusters that were functionally matched to stable plaque clusters (i.e., UP-11, UP-22 and UP-32; Fig. 2d,e, Additional file 5: Fig S4, and Additional file 4: Table S3). This indicates that the biological processes represented by these clusters are highly activated in unstable compared to stable plaques. Although other unstable and stable plaque clusters also showed significant gene overlap (Additional file 5: Fig S3c), they did not map to meaningful biological processes based on GSOA, leaving their functional relevance unclear. Consequently, these three clusters were selected for further investigation in the following analysis.

### T cell cluster emerging as an important driver in plaque phenotype transition

**Fig. 3:**
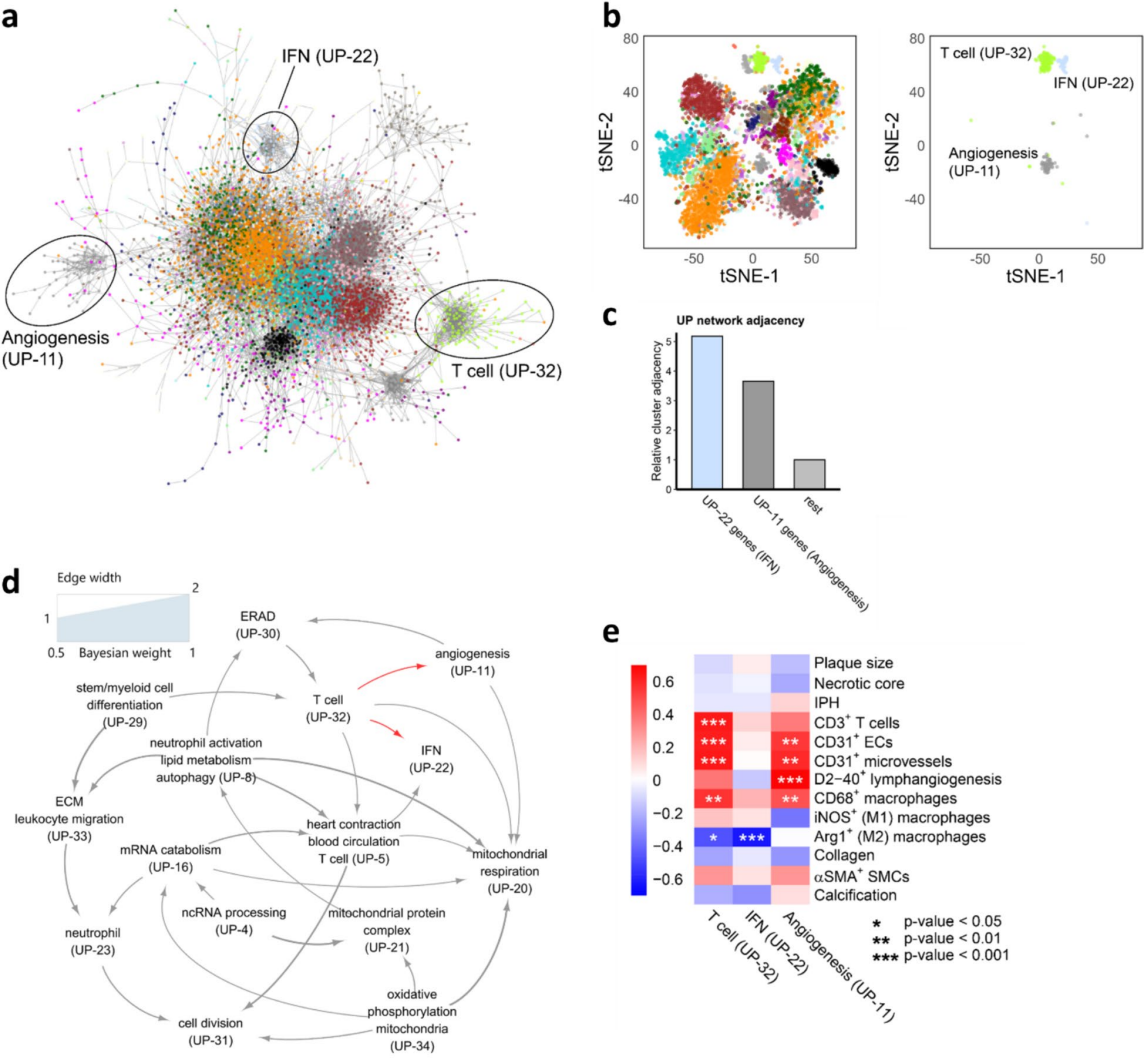
Analyses of the unstable plaque WGCNA co-expression network. **a** Visualization of the unstable plaque co-expression network, where genes are color-coded according to their WGCNA cluster. Edge weights, derived from the unstable plaque WGCNA adjacency matrix, highlight connection between genes, with only edges exceeding a weight of 0.3 displayed. This results in a network representation of 5370 out of 12,341 genes. **b** t-SNE plots of the genes from Fig 3a based on their expression profiles in unstable plaques. The left panel shows clusters colored according to the 38 unstable plaque WGCNA clusters, while the right panel focuses on three principal clusters (UP-11, *n* = 62; UP-22, *n* = 45; UP-32, *n* = 111). **c** Bar plot depicting the relative average adjacency of the UP-11 cluster, UP-22 cluster, and remaining clusters (“rest”) to the T cell-specific cluster (UP-32) based on the WGCNA adjacency matrix of the unstable plaque network. The average adjacency between UP-32 and all other clusters was normalized to 1 and used as the reference. **d** Directed Bayesian network illustrating the relationships among the 15 most biologically meaningful UP WGCNA clusters (CES > 3). **e** Heatmap showing correlations and significance levels between eigengenes of the three unstable plaque co-expression clusters and plaque traits assessed by IHC. Adj – Adjusted, Arg – Arginine, ECM – Extracellular matrix, ERAD – Endoplasmic reticulum–associated protein degradation, IFN – Interferon, IHC - Immunohistochemistry, (iNOS – Nitric oxide synthases, mRNA – messenger ribonucleic acid, ncRNA – non-coding ribonucleic acid, NES – normalized enrichment score, SMA – Smooth muscle actin, SMC – Smooth muscle cells, SP – stable plaque, t-SNE – t-distributed stochastic neighbor embedding, UP – unstable plaque, WGCNA - Weighted Gene Co-expression Network Analysis

To examine changes occurring during the transition from stable to unstable plaques, we analyzed the biologically relevant unstable plaque networks in detail. The network genes were found to be both highly interconnected and clustered, as shown in the network visualization and t-SNE plots (Fig. 3a,b). Notably, three functional clusters (“T cell,” “IFN,” and “angiogenesis”) were clearly separated from the main network cluster. Since the downscaled network and t-SNE plots did not effectively show cluster proximities, the average distance between clusters was calculated. The “T cell” cluster (UP-32) was the central point, being preserved in both unstable and stable plaques (Fig. 2c) and strongly dysregulated in the unstable plaque phenotype compared to the stable plaque phenotype (Additional file 4: Table S3). Based on the unstable plaque network adjacency, the “T cell” cluster (UP-32) was found to be nearly five times closer to the “IFN” cluster (UP-22) and more than 3 times closer to the “angiogenesis” cluster (UP-11) compared to all other clusters (rest) (Fig. 3c). Notably, in the stable plaque network, these (co-expressed) subnetworks were much more distant, highlighting the relevance (and specificity) of cross-interaction of these subnetworks for unstable plaques (Additional file 5: Fig S5). The proximity analysis suggested that UP-32 is close to UP-22, a finding that is also consistent with their relative positioning in the hierarchical clustering dendrogram (see red clusters in the dendrogram in Additional file 5: Fig S2a). These results suggest that the unstable plaque-specific “T cell,” “IFN,” and “angiogenesis” clusters are closely interconnected and likely interact at a transcriptional or functional level within the unstable plaque.

To explore the hierarchy and direction of the interactions between clusters within the gene network, a Bayesian network model was constructed for the 15 most biologically meaningful unstable plaque clusters (CES > 3) (Fig. 3d). The model shows the “T cell” cluster as a central hub, positioned upstream of the “angiogenesis” and “IFN” clusters (indicated by red arrows), which are themselves upstream of the “mitochondrial activity” clusters (UP-20, −21, and −34) and the “cell division” cluster (UP-31), which ranks lowest. Notably, the three mitochondria-related clusters (UP-20, −21, and −34) are closely grouped in the dendrogram (Additional file 5: Fig S2a, blue clusters) and directly linked in the Bayesian network (Fig. 3d), underpinning the validity of the causal network. These findings point to a pivotal role of the “T cell” cluster in unstable plaques. Histological analysis of adjacent plaque sections showed strong correlations between the “T cell” and “angiogenesis” clusters and key plaque traits, assessed through CD3^+^ T cell and CD31^+^ endothelial cell staining’s, supporting both the predicted biological roles of these clusters and their potential functional interactions (Fig. 3e). Although the IFN-related UP-22 module genes were enriched in plaques transitioning toward an unstable state (Fig. 2e), the IFN cluster, as a whole, did not show significant correlations with intraplaque hemorrhage or other unstable-associated plaque traits (Fig. 3e). Thus, IFN-related genes of UP-22 mainly mark the transition to an unstable plaque state. This could proceed in a hemorrhage-independent manner, or its activity may already plateau at very low levels of hemorrhage (once IPH is established).

Collectively, the above findings indicate that the “T cell,” “IFN,” and “angiogenesis” clusters form a tightly interconnected network in unstable plaques. Moreover, Bayesian network analysis further identified the “T cell” cluster as the key driver in plaque destabilization, upstream of clusters like “IFN” and “angiogenesis,” leading to the selection of the “T cell” cluster for further study.

### The regulation of the T cell-specific cluster

**Fig. 4:**
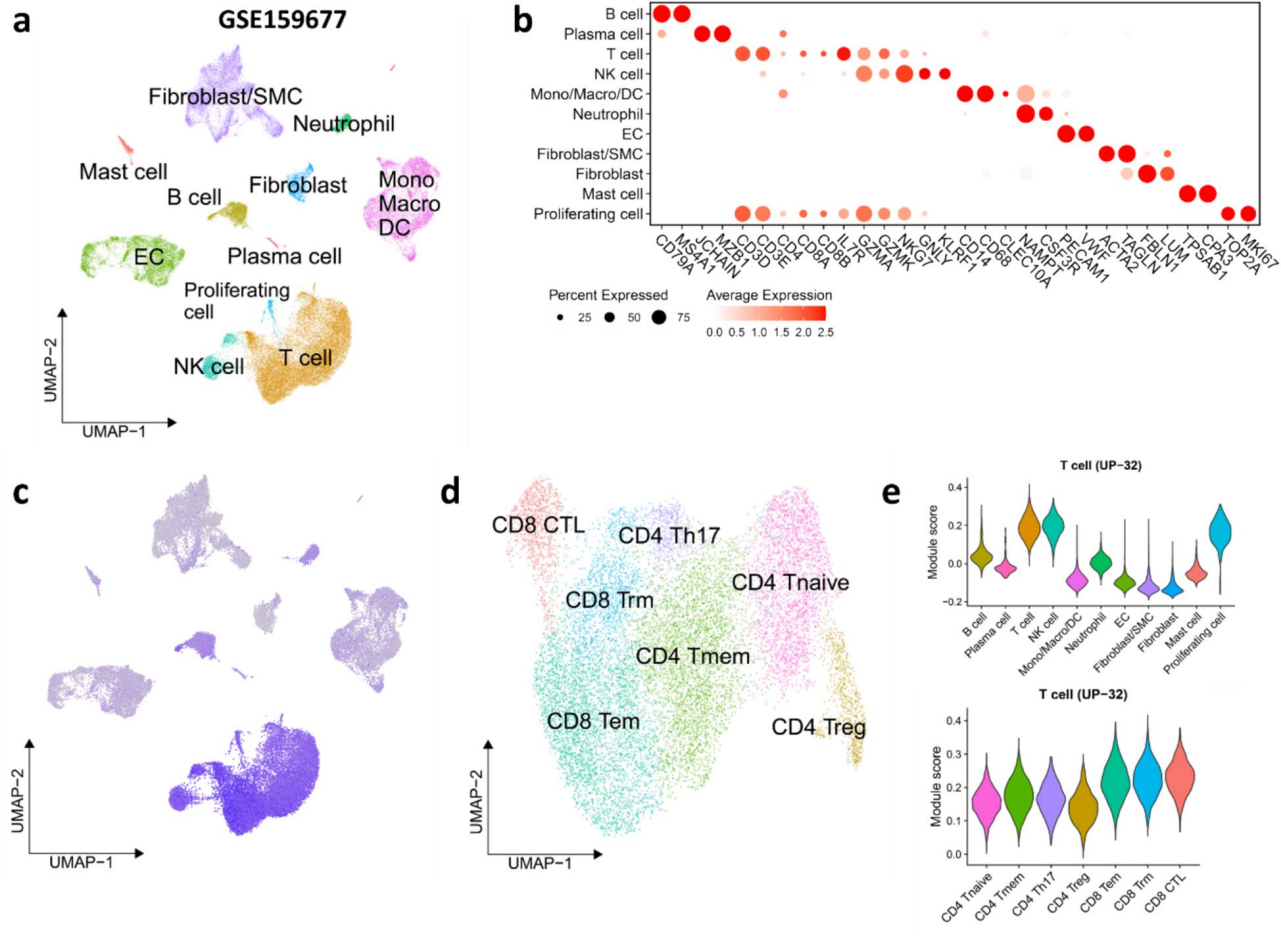
UP-32 gene co-expression cluster is enriched in plaque T cells (GSE159677). **a** UMAP plot shows cells from the carotid atherosclerotic core and patient-matched proximal adjacent tissues (both ***n*** = 3) in GSE159677 dataset. **b** Dot plot shows the markers of each cell type in **a**. **c** UMAP plot shows the enrichment of the UP-32 T cell-specific cluster (UP-32; ***n*** = 250) in cells from GSE159677. d UMAP plot shows the subtypes of plaque T cells in GSE159677. e Violin plot shows the enrichment of the UP-32 T cell-specific cluster in plaque cells from GSE159677 dataset. Mono – monocyte, Macro – macrophage, DC – dendritic cell, EC – endothelial cell, NK – natural killer, SMC – smooth muscle cell, Tnaive – naïve T cell, Tmem – memory T cell, Tem – effector memory T cell, Trm – resident memory T cell, CTL – cytotoxic T cell, Treg – regulatory T cell, Th17 – T helper 17 cell

We next examined the expression of gene members of UP-32, the “T cell” cluster, using an independent human carotid plaque scRNA-seq dataset from a comparative paired analysis of atherosclerotic core and proximally adjacent region of three asymptomatic patients with type VII calcified plaques (GSE159677) [22] and assigned the cell clusters to the respective cell types, based on their expression signature (Fig. 4a,b). Consistent with the GO term enrichment, UP-32 cluster genes were predominantly expressed by plaque T cells (Fig. 4c). Focusing on T cells specifically (Fig. 4d), and mapping subclusters to lymphocyte subsets, the highest module scores were observed in CD8^+^ effector memory (CD8^+^ Tem), CD8^+^ resident memory (Trm), and CD8^+^ effector (CTL) T cells, alongside NK cells and proliferating lymphocytes (Fig. 4e). In fact, several UP-32 cluster genes (i.e., *CCL5*, *GZMA*, *NKG7*, *CST7*, *GZMK*, *GZMM*, *PYHIN1*, *KLRG1*, *CTSW*, *GZMB*, *KLRD1*, *PRF1*) are known markers for CD8^+^ T cell subpopulations such as Tem, Tm, and CTL [51–53]. Supporting this, analysis of another human plaque scRNA-seq dataset from Giannarelli et al. (GSE224273) [13, 24, 25] similarly indicated strong alignment between the “T cell” cluster UP-32 and plaque CD8^+^, CD4^+^, and NK cell populations (Additional file 5: Fig S6a-d).

**Fig. 5:**
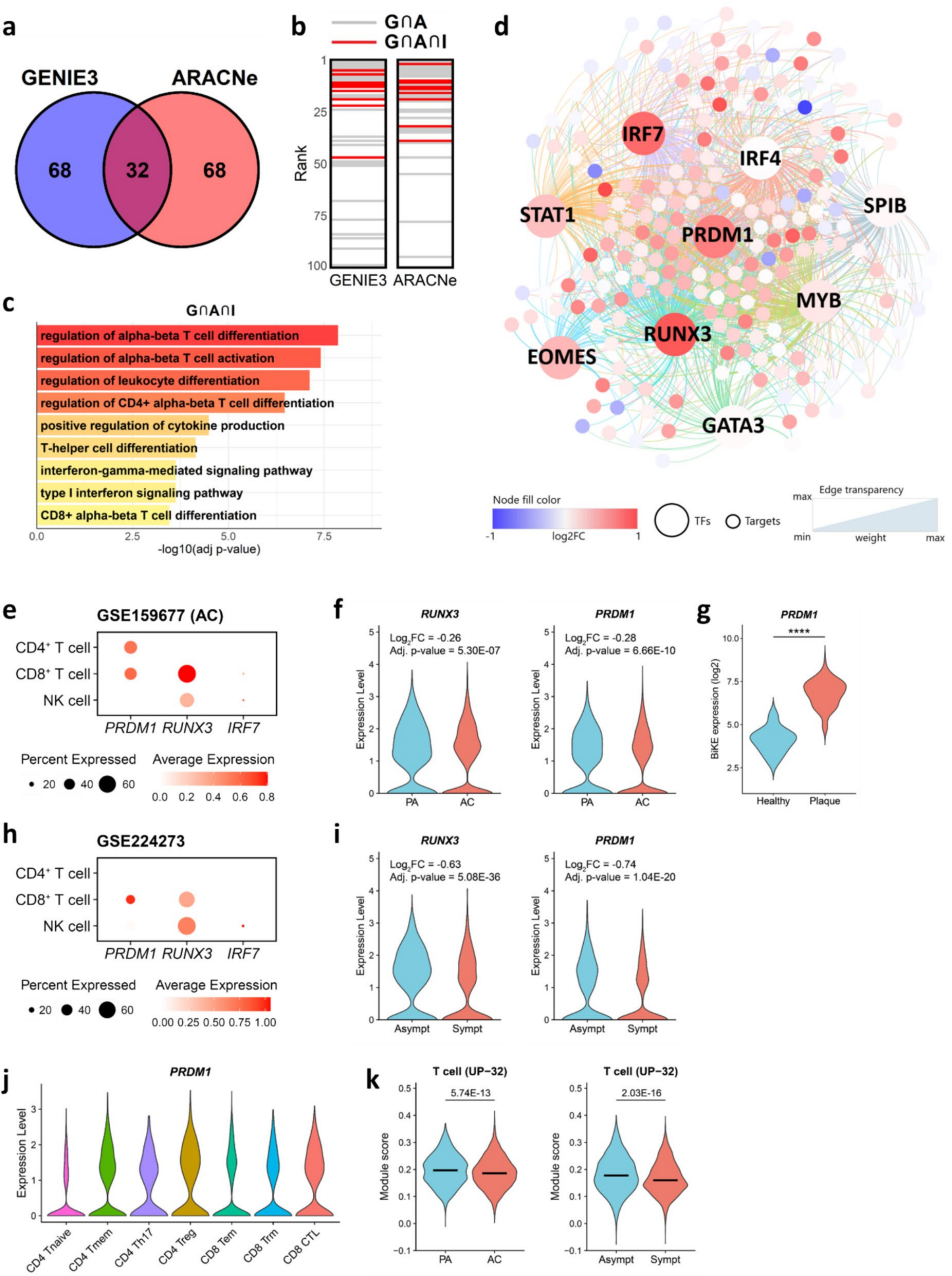
Regulatory network reconstruction for UP-32 T cell-specific cluster. **a** Venn diagram illustrating the overlap among the top 100 ranked TFs identified by GENIE3 and ARACNe algorithms. **b** Ranking positions of the 32 overlapping TFs in Fig 5a in the top 100 TFs lists of GENIE3 and ARACNe. TFs that intersect with iRegulon results are marked with red lines (9 TFs, G⋂A⋂I), while those exclusive to GENIE3 or ARACNe are marked with gray lines (23 TFs, G⋂A). ⋂ – intersection symbol. **c** Bar plot showing the GSOA results for the nine TFs identified in Fig 5b. The top nine representative significant GO terms are shown. **d** Regulatory network visualization of genes in T cell-specific cluster (UP-32; *n* = 195 of 250 genes) predicted to be regulated by the nine TFs from Fig 5b as determined by iRegulon. Each TF-target network is color-coded, with gene color representing log2 fold change (log2FC; unstable vs. stable plaques) of gene expression in unstable compared to stable plaques and edge transparency reflecting GENIE3 weight. TFs are presented by large circles, and target genes by small globes. **e** Dot plots show the expression level of *PRDM1*, *RUNX3*, and *IRF7* in CD4^+^, CD8^+^ T cells, and NK cells in atherosclerotic core tissues (AC) from GSE159677 dataset. **f** Violin plots show the expression of *PRDM1* and *RUNX3* between proximally adjacent (PA) and atherosclerotic core (AC) tissues based on GSE159677 dataset. **g** Violin plots show the expression of *PRDM1* in carotid plaques from *n* = 127 patients and *n* = 10 control/healthy arteries. **h** Dot plots show the expression level of *PRDM1*, *RUNX3*, and *IRF7* in CD4^+^, CD8^+^ T cells, and NK cells in carotid plaques from GSE224273 dataset. **i** Violin plots show the expression of *PRDM1* and *RUNX3* between asymptomatic and symptomatic patients based on GSE224273 dataset. **j** Violin plot shows the expression of *PRDM1* across T cell subsets based on GSE159677. **k** Violin plots show the enrichment of the UP-32 T cell cluster in plaque T cells from GSE159677 (left) and GSE224273 (right) dataset, with median level indicated per group. *P*-values were calculated based on the Wilcoxon rank-sum test, with Benjamini-Hochberg correction for multiple testing. AC – atherosclerotic core, GO – gene ontology, GSOA – Gene Set Ontology Analysis, PA – proximal adjacent, TFs – transcription factors

Co-expression of cluster genes suggests a shared regulatory framework. To explore this, we constructed GRNs using GENIE3 [36] and ARACNe [37] to identify key transcription factors (TFs) potentially regulating UP-32 genes in human atherosclerotic plaque. From these GRNs, we selected the top 100 TFs with the highest connectivity to UP-32 genes. By intersecting the results of both analyses, we identified 32 top TFs that are common in both networks (Fig. 5a). Next, we integrated these results with knowledge-driven motif-prediction analysis from iRegulon [40] to refine the list of cluster-relevant TFs. Nine of the 32 TFs ranked highly in both the intersect analysis and iRegulon predictions (Fig. 5b) and all were involved in CD4^+^ and CD8^+^ αβ (naïve) T cell activation (Fig. 5c), reinforcing the T cell-specific signature of the UP-32 cluster.

Among the selected TFs, IRF7, PRDM1, and RUNX3 were highly upregulated in unstable versus stable plaques. Three other TFs (STAT1, EOMES, and MYB) were only moderately upregulated in unstable plaques. We then constructed an integrated gene regulatory network for UP-32, the “T cell” cluster, incorporating the nine top TFs (Fig. 5d). While all top TFs showed a strong connection to the UP-32 hub genes, RUNX3, PRDM1, and IRF7 emerged as key regulators, and connected strongly to the central hub genes. Overall, our study shows significant activation of a T cell-associated gene regulatory network in unstable plaque, primarily driven by PRDM1, RUNX3, IRF4, and IRF7, with PRDM1, RUNX3, and IRF7 showing dysregulation in unstable compared to stable plaques in our cohort.

Our bulk microarray dataset lacks the resolution to conclusively differentiate between cell populations and their gene expression profiles. To overcome this limitation, we examined the expression of the most dysregulated TFs (PRDM1, RUNX3, and IRF7) using scRNA-seq datasets from Alsaigh et al. (GSE159677) and Giannarelli et al. (GSE224273) [13, 22–25]. The Alsaigh dataset includes gene expression data from over 50,000 cells from carotid artery tissue of CEA patients, comparing plaque segments with proximal non-atherosclerotic segments (*n* = 3 for each group). The Giannarelli dataset captures expression profiles from 10,000 cells in CEA specimens (2 symptomatic, 4 asymptomatic). Our analysis of the dataset from Alsaigh et al. [22] demonstrates that unlike *IRF7*, the expression of *RUNX3* and *PRDM1* is highly specific to plaque T cells (Fig. 5e). Moreover, both *RUNX3* and *PRDM1* were significantly downregulated in CEA plaque T cells versus T cells from proximal adjacent tissues (*PRDM1*: log_2_FC = − 0.28, Adj. *p*-value = 6.66E − 10; *RUNX3*: log_2_FC = − 0.26, Adj. *p*-value = 5.30E − 07) (Fig. 5f). The association of PRDM1 with atherosclerosis was further underpinned by analysis of an independent human CEA plaque cohort (BiKE [21]), showing almost ten fold higher expression of *PRDM1* (P < 0.0001) in atherosclerotic (*n* = 127) than in control non-atherosclerotic artery tissue (*n* = 10) (Fig. 5g). Furthermore, analysis of Giannarelli’s dataset [13, 24] confirmed T cells as main source of *RUNX3* and *PRDM1* expression (Fig. 5h) and indicated that expression of both *RUNX3* and *PRDM1* was significantly downregulated in T cells in plaque of symptomatic compared to asymptomatic patients (*PRDM1*: log2FC = − 0.74, Adj. *p*-value = 1.04E − 20; *RUNX3*: log2FC = − 0.63, Adj. *p*-value = 5.08E − 36) (Fig. 5i), while *IRF7* remained unaffected (data not shown). Notably, *PRDM1* did not exhibit subset-specific expression among T cells (albeit that PRDM1 tended to be slightly higher in CD4^+^ Treg), suggesting a broad regulatory role for this TF within the plaque T cell population (Fig. 5j). Nevertheless, the overall expression of genes from the UP-32 T cell cluster was significantly reduced in plaque T cells from the atherosclerotic core compared to the proximal (non-atherosclerotic) carotid artery region, and from symptomatic compared to asymptomatic patients, consistent with the observed downregulation of *PRDM1* (Fig. 5k).

### The regulatory role of T cell *Prdm1* in murine lesion development

**Fig. 6:**
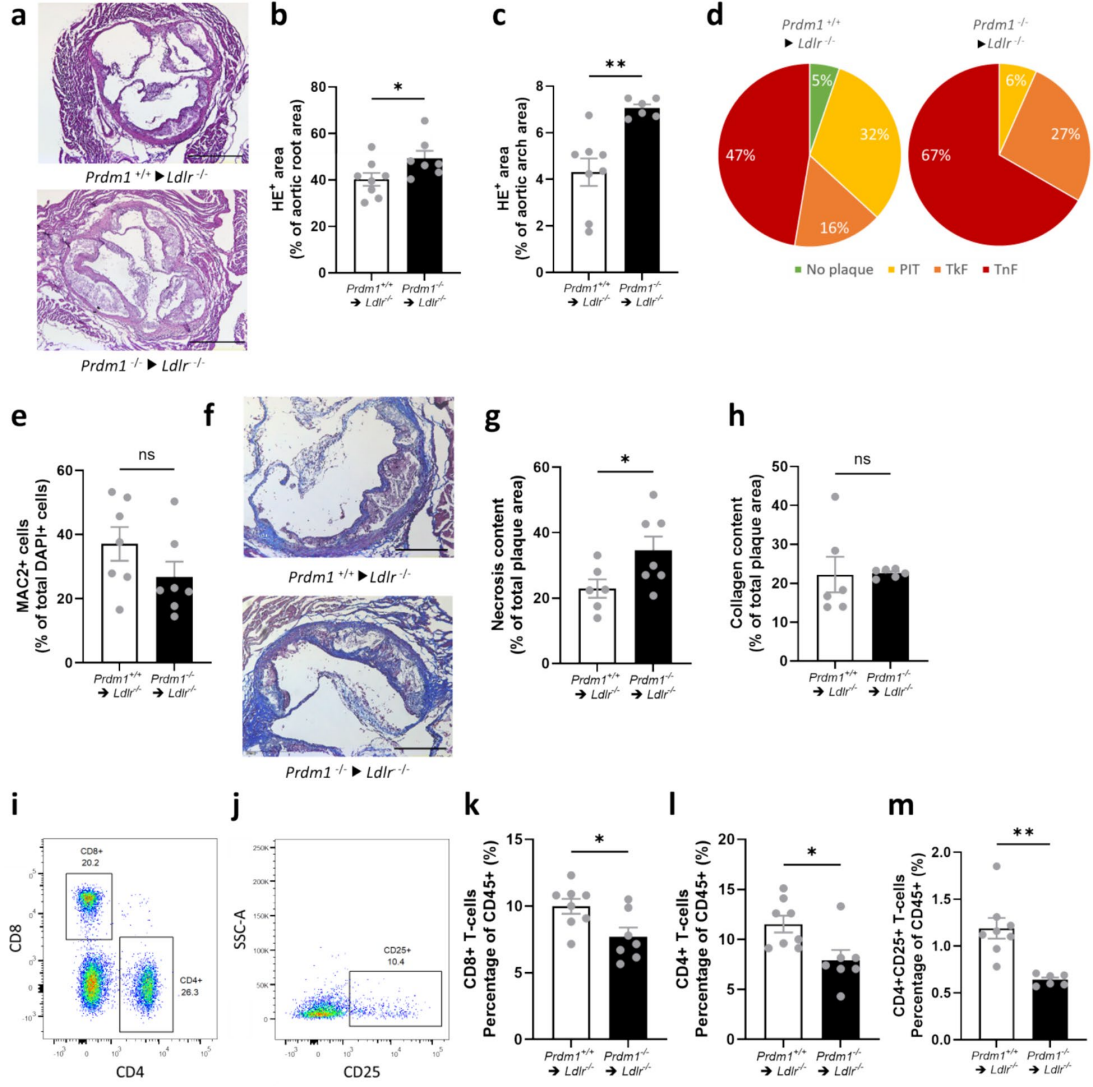
T cell-specific *Prdm1* deficiency increases lesion development. **a–c** Representative pictures of H&E staining the aortic root (a; Scale bar = 500 µm) and quantification of lesion area in aortic roots (**b**) and arches (**c**) of *Prdm1*^+*/*+^*Ldlr*^*–/–*^ and *Prdm1*^*−/−*^*Ldlr*^*–/–*^ mice following 12 weeks on a Western-type diet (WTD). **d** Classification of plaque stage as early progressive (pathologic intimal thickening, PIT), advanced with a thick fibrotic cap (TkF), or advanced with a thin fibrotic cap (TnF). **e** Quantification of macrophage in aortic root lesions, assessed by Mac2 immunofluorescent staining. **f–h** Representative pictures of Trichrome staining of the aortic roots (**f**; scale bar = 250 µm). Quantification of necrotic core area (anucleated area) (**g**) and collagen content (**h**) in aortic root lesions. **i–m** Representative flow cytometry dot plots (**i, j**) and quantification of CD8^+^ (**k**) and CD4^+^ (**l**) T cells (pre-gated: CD45^+^CD115^**−**^Gr1^**−**^B220^**−**^) and CD4^+^CD25^+^ cells (**m**) in blood. Data are presented as mean ± SEM; *n* = 6–7. Statistical significance was assessed by Student *t* test with Welch correction, Mann–Whitney test, or chi-square, as appropriate. *P < 0.05; **P < 0.01

Our network analysis identified PRDM1, RUNX3, and IRF7 as key regulators of a T cell network in unstable human atherosclerotic plaques. To further validate their role in lesion development, we turned to a mouse model of atherosclerosis. Given that IRF7 was not specific for plaque T cells in two datasets (Fig. 5e, h, Additional file 5: Fig S7) and had been previously implicated in atherosclerosis by influencing macrophage inflammation and lipid homeostasis [54], we excluded it from further analysis. Since RUNX3 is reported to exert much of its T cell regulatory function through PRDM1 [55], we focused on the role of T cell PRDM1 in atherosclerosis. Interestingly, *PRDM1* was downregulated in T cells in plaques of symptomatic compared to asymptomatic patients (Fig. 5i), suggesting it might have a protective role in plaque destabilization. To investigate this, we induced atherosclerosis in *Ldlr*^–*/*–^ mice stably reconstituted with bone marrow from *CD4*^*Cre*+^*/Prdm1*^*flox/flox*^ (*Prdm1*^−/−^) or *Prdm1*^*flox/flox*^ (*Prdm1*^+*/*+^) mice and fed them a high-fat diet for 12 weeks. The CD4^Cre^ driver, known to target both CD4 and CD8, ensured *Prdm1* deficiency in both T cell lineages [47, 56]. Mice deficient in *Prdm1* showed a significant increase in lesion size in both the aortic root and arch (Fig. 6a-c). Additionally, plaques in the aortic root of *Prdm1*^−/−^*Ldlr*^−/−^ mice were significantly more advanced (< 0.0001) compared to those of the *Prdm1*^+/+^*Ldlr*^−/−^ mice (Fig. 6d), with no differences in the number of lesional macrophages (Fig. 6e). Interestingly, *Prdm1* deficiency led to a significant increase in necrotic core content but not in the collagen deposition in aortic root lesions, indicative of a more advanced plaque phenotype (Fig. 6f–h). As expected, the relative abundance of CD8^+^, CD4^+^, and CD4^+^CD25^+^ T cells was significantly reduced in blood of the *Prdm1*^−/−^ mice (Fig. 6i–m), with similar trends observed in bone marrow, lymph nodes, and spleen (Additional file 7: Table S5). Of note, total cholesterol and triglyceride levels, and body weight did not differ between *Prdm1*^−/−^ and *Prdm1*^+/+^ chimeras (Additional file 8: Table S6). In summary, this in vivo murine model strongly supports the protective role of T cell-specific PRDM1 in atherosclerosis, with *Prdm1* deficiency leading to larger, more advanced plaques and a reduction in T cell populations.

### In silico drug repurposing based on the unstable plaque T cell cluster

While the findings above identify the PRDM1-driven T cell cluster as a key driver of plaque progression, the poor druggability of PRDM1 and broader activity in NK and B cells disqualify PRDM1 as a viable therapeutic target. Therefore, we sought to identify drugs that are capable of targeting and deactivating this T cell network. To this end, we pursued in silico drug screening, using the LINCS L1000 dataset, which contains drug-induced gene expression profiles from the JURKAT T cell line. We screened for compounds whose transcriptional signatures inversely correlate with the dysregulation of the complete T cell cluster (UP-32) seen in unstable versus stable plaque. Connectivity scores close to + 1 indicate similar profiles, while scores near − 1 Suggest opposite profiles, reflecting the ability to Suppress activation of the network of interest. A total of 57 drugs with negative connectivity scores of < − 0.7 were considered potentially effective (Additional file 9: Table S7). Notably, among the top-ranked candidates were two epidermal growth factor receptor (EGFR) signaling blockers, which exhibited very high connectivity scores of approximately − 0.9, along with four antagonists targeting other growth factor receptor pathways (vascular endothelial growth factors (VEGFRs) and platelet-derived growth factor receptors (PDGFRs)) (Table 2). These findings further corroborate our earlier report, which demonstrated strong upregulation of the EGFR pathway in UP plaques [17].

**Table 2:** Selected drugs targeting the UP-32 T cell cluster

Score	Drug	Dose	Effect
−0,914	Dacomitinib	10.0 µM	EGFR inhibitor
−0,898	Osimertinib	10.0 µM	EGFR inhibitor, induces cell death and inhibits tumor growth in EGFR-overexpressing tumor cells
−0,863	Pazopanib	3.33 µM	Inhibitor of VEGFR1, VEGFR2, PDGFRα, PDGFRβ, KIT, FGFR3, ITK, FGF1, SH2B3, antineoplastic
−0,821	Quizartinib	0.04 µM	FLT3 and PDGFRs inhibitor, inhibits leukemic cell proliferation and apoptosis
−0,794	Tivozanib	10.0 µM	Inhibitor of VEGFRs 1, 2, and 3 with potential antiangiogenic and antineoplastic activities
−0,737	Sunitinib	0.12 µM	Inhibitor of multiple receptor tyrosine kinases including all VEGFRs and PDGFRs, inhibits angiogenesis and cell proliferation

## Discussion

Network-based analyses of high-throughput gene expression data have become essential for unraveling biological processes and regulatory mechanisms underlying human disease. Moreover, these approaches facilitate the translation of molecular discoveries into clinical applications [57]. In this study, we constructed a comprehensive network to identify key processes involved in the destabilization of atherosclerotic plaques, transitioning from a stable (low-risk) to unstable (high-risk) plaque phenotype. To achieve this, we utilized multiple computational methodologies, including gene co-expression and protein–protein interaction networks, Bayesian inference, regulatory network construction, and differential expression analyses, on transcriptomic data from the MaasHPS cohort [18]. Our findings uncovered a co-expressed gene cluster with a strong T cell signature that plays a critical role in the progression from stable- to unstable-risk plaques, potentially mediated through interactions with IFN signaling and angiogenesis. Key regulators of this network, including PRDM1, RUNX3, and IRF7, were identified. In particular, a murine atherosclerosis model revealed that *Prdm1* deficiency in T cells resulted in significant plaque growth and enhanced necrotic core formation, while reducing T cell numbers. Finally, in silico drug repurposing revealed EGFR inhibition as a potentially powerful target for intervention in this detrimental network in human atherosclerotic plaque.

While traditional transcriptional studies often focus on differential gene expression followed by pathway enrichment, we adopted a network-centric approach to capture complex inter-gene interactions and co-regulated pathways relevant to plaque destabilization. By constructing a Bayesian network, we identified the directionality and interplay between co-expressed gene clusters and their associated biological processes, allowing us to distinguish upstream from downstream events. This approach improves candidate prioritization and is broadly applicable to any gene expression dataset containing multiple study arms.

Our WGCNA and Bayesian network analysis revealed that T cell activity is a key modulator of IFN (types I and II) signaling and angiogenesis within unstable plaques, consistent with previous reports showing increased T cell content in advanced atherosclerotic plaques [58]. Interestingly, the T cell cluster from stable plaques (SP-7), which contained 3403 genes, showed broader and more diffuse involvement in immune processes, likely reflecting the lower immune cell density in stable plaques. In contrast, the unstable plaque-derived T cell cluster (UP-32) exhibited stronger influence over downstream immune gene modules, notably the “IFN” and “angiogenesis” modules. Historically, CD4^+^ T cells were thought to be the dominant T cell subset in atherosclerosis [59], but more recent studies, including deep histological examination [58], mouse model research (for reviews see [60, 61]), and scRNA-seq studies [13, 24, 62] highlight the critical role of CD8^+^ T cells in atherosclerosis. CD8^+^ T cells in plaques, especially in symptomatic patients, exhibit signs of activation and exhaustion [13]. Our T cell cluster signature was mapped to both CD4^+^ (naïve and memory CD4^+^ T cells) and CD8^+^ subsets (e.g., effector memory, memory, and cytotoxic CD8^+^ T cells), with a stronger presence in CD8^+^ T cells.

Further exploration of the gene signature of the “T cell” module, UP-32 cluster, through GRN analysis identified PRDM1, RUNX3, IRF7, and EOMES, and to a lesser extent MYB and IRF4 as key TFs. PRDM1, EOMES, and IRF7 are critical for effector and memory CD8^+^ T cell function, including IFN-γ, perforin and granzyme secretion [63], and T-cell exhaustion [64], and are implicated in T cell exhaustion. On the other hand, STAT1 and GATA3 underscored the role of CD4^+^ T helper (Th) 1 and Th2 cells, respectively. Interestingly, several of these transcription factors, including RUNX3 [65], STAT1 (for a review see [66]), IRF7 [54], and the Th2 hallmark GATA3 [67], had already been implicated in lesion formation in experimental models of atherosclerosis. The association of the “T cell” module with high-risk plaque and its regulatory context suggests that CD4^+^ and especially CD8^+^ T cells are key modulators of gene networks contributing to plaque destabilization and vulnerability.

Previous research highlights the dual role of T cells in atherosclerosis, where certain subsets, particularly CD8^+^ T cells can be either atherogenic or atheroprotective [67]. For example, deficiencies or compromised functioning of Tregs or negative T cell regulation like CBL-B have been shown to accelerate plaque formation in mouse models [68, 69]. Our study aligns with these findings, showing that the PRDM1-driven gene module plays a protective role in atherosclerosis. T cell-specific *Prdm1* deficiency led to reduced circulating T cell numbers, including Tregs, accelerated plaque development, and increased plaque destabilization. Considering however the broader activity profile of PRDM1, combined with the poor druggability of TFs in general, PRDM1 is unlikely to qualify as a suitable drug target. Therefore, we pursued a genomics network-guided drug screening approach which revealed several drugs, with EGFR inhibitors as main candidates, which do not suffer from these pitfalls, and may have potential in plaque stabilizing therapy. Interestingly, both anti-VEGF/VEGFR and EGFR inhibition have already been shown to attenuate atherosclerosis in a mouse model of disease, suggestive of their promise in ASCVD therapy [70–73]. Moreover, CD4^+^ T cell-specific EGFR ablation was shown to lead to T cell anergy and to reduce plaque T cell infiltration and atherosclerosis in *Ldlr*^*−/−*^ mice, highlighting the role of T cells in this process [73]. Our study adds to this notion and underpins the relevance of EGFR for human disease.

However, this study has some limitations that should be considered. The MaasHPS cohort consists of symptomatic male patients, which may restrict broader applicability [18]. Nevertheless, our validation using scRNA-seq datasets from a mixed-gender cohort demonstrates clear PRDM1 dysregulation, suggesting the findings are not sex-specific [13, 24]. Additionally, our mouse model does not fully represent late-stage symptomatic atherosclerosis, so the role of PRDM1 in advanced disease remains uncertain.

## Conclusion

In conclusion, we identified a T cell-driven gene network linked to plaque destabilization, modulating IFN and angiogenesis signaling, and is mainly orchestrated by PRDM1. This network offers a promising therapeutic target for atherosclerosis intervention. While PRDM1 is poorly druggable, our study identifies EGFR inhibitors as potential candidate drugs able to target this key network in a coordinate manner, and suppressing culprit immune and angiogenic pathways in atherosclerotic plaque destabilization.

## Supplementary Information

Additional file 1: Materials.

Additional file 2: Table S1-1. Patient information MaasHPS dataset. Table S1-2. Patient information BiKE dataset.  

Additional file 3: Table S2-1. GSOA of WGCNA clusters (SP). Table S2-2. GSOA of WGCNA clusters (UP).

Additional file 4: Table S3-1. GSEA of WGCNA clusters (UP). Table S3-2. GSEA of WGCNA clusters (UP; differential expression of GSEA core genes).

Additional file 5: Fig S1-S7 - Fig S1. Plaque stage classification. Representative image of the classification of the plaques stage in the aortic root: I - early progressive (pathologic intimal thickening, PIT); II - advanced with a thick fibrotic cap (TkF); III - advanced with a thin fibrotic cap (TnF). Scale bar = 500 µm. Fig S2. Additional results of unstable plaque WGCNA co-expression network. (a-b) Hierarchical clustering based on the eigengenes of unstable plaque WGCNA clusters (a) and stable plaque WGCNA clusters (b), shown as dendrograms. Fig S3. Comparison between stable and unstable WGCNA clusters. (a) For the top 10 stable co-expression clusters, the significance levels of the top-ranked overrepresented GO terms per cluster were visualized as a dot plot. (b) Venn diagrams showing gene overlap between selected stable and unstable clusters. (c) Heatmap shows Jaccard Index between all stable and unstable clusters. The number in square brackets indicates the size of the cluster. Four pairs of stable and unstable clusters highlighted are selected for the Venn Diagram visualization in (b). Significance level in (b-c) was evaluated by hypergeometric testing. Fig S4. Heatmaps of the unstable WGCNA clusters. Heatmaps showing the expression of genes in UP-32, UP-22, and UP-11 between unstable and stable plaques. For clarity sake, only core enrichment genes contributed to GSEA are shown. Fig S5. Relative cluster adjacency in stable plaques. Bar plot depicting the relative average adjacency of the UP-11 cluster, UP-22 cluster, and remaining clusters (“rest”) to the T cell-specific cluster (UP-32) based on the WGCNA adjacency matrix of the stable plaque network. The average adjacency between UP-32 and all other clusters was normalized to 1 and used as the reference. Fig S6. UP-32 gene co-expression cluster is enriched in plaque T cells (GSE224273). (a) UMAP plot shows carotid plaque cells from asymptomatic (n = 2) and symptomatic patients (n = 4) in GSE224273 dataset. (b) Dot plot shows the markers of each cell type in (a). (c) UMAP plot shows the enrichment of the UP-32 T cell-specific cluster (UP-32; n = 250) in cells from GSE224273 dataset. (d) Violin plot shows the enrichment of the UP-32 T cell-specific cluster in plaque cells from GSE224273 dataset. EC– endothelial cell, NK – natural killer, SMC – smooth muscle cell. Fig S7. Expression of PRDM1, RUNX3 and IRF7 in plaque scRNA-seq datasets. (a) UMAP plots show cells from the atherosclerotic core (AC) and proximal adjacent (PA) tissues in GSE159677 dataset and (b) cells from the asymptomatic and symptomatic patients in GSE224273 dataset. (c-d) UMAP plots show the expression of PRDM1, RUNX3, and IRF7 in GSE159677 dataset (c) and GSE224273 dataset (d).

Additional file 6: Table S4-1. Gene overlap between SP and UP clusters, evaluated by Jaccard Index. Table S4-2. Gene overlap between SP and UP clusters, evaluated by hypergeometric testing.

Additional file 7: Table S5. Frequency of T cells in the inguinal lymph node, spleen and bone marrow of mice fed with HFD for 12 weeks.

Additional file 8: Table S6. Cholesterol and triglycerides levels and body weight of mice fed with HFD for 12 weeks.

Additional file 9: Table S7. Drug repurposing results based on the gene expression in the T cell cluster UP-32.

## Data Availability

MaasHPS transcriptomics can be accessed from the Gene Expression Omnibus (GEO) with accession number GSE163154 [18]. BiKE microarray data has been deposited at NCBI Gene Expression Omnibus and is publicly available with accession number GSE21545 [21]. Human plaque scRNA-seq data used in this study were downloaded from GEO with accession numbers GSE159677 [23] and GSE224273 [25], respectively. Scripts used to reproduce the main results of this study are available at the GitHub repository ( [https://github.com/jha14/PRDM1\_Tcell\_atherosclerosis](https:/github.com/jha14/PRDM1_Tcell_atherosclerosis) ) [74]. Processed data objects are deposited in Zenodo ( [https://doi.org/10.5281/zenodo.15852128 ](https:/doi.org/10.5281/zenodo.15852128 ) ) [75].

## Abbreviations

AC: Atherosclerotic core
Adj: Adjusted
ADT: Antibody-Derived Tag
ADT/HTO: Antibody-Derived Tag/Hashtag Oligonucleotide
AHA: American Heart Association
ARACNe: Algorithm for the Reconstruction of Accurate Cellular Networks
Arg: Arginine
BiKE: Biobank of Karolinska Endarterectomies
CCD: Charge-coupled device
CD: Cluster of Differentiation
CEA: Carotid endarterectomy
CES: Cluster Enrichment Score
CITE-seq: Cellular Indexing of Transcriptomes and Epitopes by Sequencing
CTL: Cytotoxic T cell
DAPI: 4',6-Diamidino-2-Phenylindole
DC: Dendritic cell
EC: Endothelial cell
ECM: Extracellular matrix
ECs: Endothelial cells
EDTA: Ethylenediaminetetraacetic acid
EGFR: Epidermal growth factor receptor
ERAD: Endoplasmic reticulum–associated protein degradation
FACS: Fluorescence-activated cell sorting
FC: Fold change
GENIE3: Gene Network Inference with Ensemble of Trees
GO: Gene ontology
GRN: Gene regulatory network
GSE: Gene Expression Omnibus Series
GSEA: Gene set enrichment analysis
GSOA: Gene set overrepresentation analysis
H&E: Hematoxylin–eosin
HGNC HUGO: Gene Nomenclature Committee
HTO: Hashtag oligonucleotide
IFN: Interferon
IG: Immunoglobulin
IHC: Immunohistochemistry
iNOS: Nitric oxide synthases
IPH: Intraplaque hemorrhage
Ldlr^-/-^: Low-density lipoprotein receptor knockout mouse
LINCS L1000: Library of Integrated Network-Based Cellular Signatures
M1-like Macrophage: Classically Activated Macrophage (iNOS Positive)
M2-like Macrophage: Alternatively Activated Macrophage (Arg1 Positive)
MaasHPS: Maastricht Human Plaque Study
Mac2: Macrophage Antigen-2
Macro: Macrophage
Mono: Monocyte
MPTC: Maastricht Pathology Tissue Collection
mRNA: Messenger ribonucleic acid
NCBI: National Center for Biotechnology Information
ncRNA: Non-coding ribonucleic acid
NK: Natural killer
PA: Proximal adjacent
PBS: Phosphate-buffered saline
PDGFR: Platelet-derived growth factor receptor
Prdm1: PR Domain Zinc Finger Protein 1
RIN: RNA Integrity Number
RNA-seq: RNA sequencing
RSN: Robust spline normalization
scRNA-seq: Single-cell RNA sequencing
SD: Standard deviation
SEM: Standard error of the mean
SMC: Smooth muscle cell
SP: Stable plaques
Tem: Effector memory T cell
TF: Transcription factor
Th: T helper cell
TkF: Thick fibrotic cap
Tmem: Memory T cell
TnF: Thin fibrotic cap
TOM: Topological overlap matrix
TR: T cell receptor
αSMA + SMCs: Alpha-Smooth Muscle Actin Positive Smooth Muscle Cells
Treg: Regulatory T cell
Trm: Resident memory T cell
VEGFR: Vascular endothelial growth factor receptor
t-SNE: T-Distributed Stochastic Neighbor Embedding
UMAP: Uniform manifold approximation and projection
UP: Unstable plaques
VSMCs: Vascular smooth muscle cells
WGCNA: Weighted Gene Co-expression Network Analysis
WTD: Western-type diet
