# Supplementary material for: Identification of a PRDM1-regulated T cell network to regulate atherosclerotic plaque inflammation
Source: Genome Med. 2025 Oct 2;17:109. doi: 10.1186/s13073-025-01541-6 (PMC12490039; doi:10.1186/s13073-025-01541-6)
Supplement: Supplementary file 1 — Additional file 1: Materials. [file 13073_2025_1541_MOESM1_ESM.docx]

**Materials**

Key resources table

| REAGENT or RESOURCE | SOURCE | IDENTIFIER |
| --- | --- | --- |
| **Antibodies** | | |
| Monoclonal anti-CD45 antibody (30-F11), APC-eFluor™ 780 | eBioscience™ | Cat#47-0451-82;  AB_1548781 |
| Monoclonal anti-CD115 antibody (AFS98), Alexa Fluor™ 488 | eBioscience™ | Cat#53-1152-82; AB_2016696 |
| Monoclonal anti-Ly-6G/Ly-6C (Gr1) antibody (RB6-8C5), Brilliant Violet 510™ | Biolegend | Cat#108438;  AB_2562214 |
| Monoclonal anti-CD11b antibody (M1/70), PE-Cyanine7 | eBioscience™ | Cat#25-0112-82;  AB_469588 |
| Monoclonal anti- CD45R (B220) antibody (RA3-6B2), eFluor™ 450 | eBioscience™ | Cat#48-0452-82;  AB_1548761 |
| Monoclonal anti-CD3e antibody (145-2C11), PerCP-Cyanine5.5 | eBioscience™ | Cat#45-0031-82;  AB_1107000 |
| Monoclonal anti-CD4 antibody (GK1.5), APC | eBioscience™ | Cat#17-0041-82; AB_469320 |
| Monoclonal anti-CD8 antibody (53-6.7), PE | eBioscience™ | Cat#12-0081-82;  AB_465530 |
| anti-Mac2 (Galectin-3) antibody (M3/38) | Cedarline | Cat#CL8942AP |
| Goat anti-Rat IgG (H+L) Cross-Adsorbed Secondary Antibody, DyLight 550 | Invitrogen | Cat#SA5-10019;  AB_2556599 |
| **Biological samples** | | |
| Carotid plaques from MaasHPS cohort | Maastricht University Medical Center+ | N/A |
| **Chemicals, peptides, and recombinant proteins** | | |
| DAPI (4',6-Diamidino-2-Phenylindole, Dihydrochloride) | Thermo Scientific | Cat#62249 |
| **Critical commercial assays** | | |
| Nucleospin RNA II kit | Macherey-Nagel GmbH & Co. KG | Cat#740955.50 |
| Illumina TotalPrep RNA Amplification Kit | Ambion, Inc. | Cat#AMIL1791 |
| Cholesterol enzymatic assays | c.f.a.s. cobas, Roche Diagnostics | Cat#3039773190 |
| Triglyceride enzymatic assays | c.f.a.s. cobas, Roche Diagnostics | Cat#4657594190 |
| **Deposited data** | | |
| MaasHPS human carotid plaque mRNA expression profile | Jin et al.[18-20] | <https://www.ncbi.nlm.nih.gov/geo/query/acc.cgi?acc=GSE163154> |
| Biobank of Karolinska Endarterectomies (BiKE) | Matic et al.[21-23] | <https://www.ncbi.nlm.nih.gov/geo/query/acc.cgi?acc=GSE21545> |
| Human carotid plaque scRNA-seq data | Alsaigh et al.[24] | <https://www.ncbi.nlm.nih.gov/geo/query/acc.cgi?acc=GSE159677> |
| Human carotid plaque scRNA-seq data | Fernandez et al.[25, 26] | <https://www.ncbi.nlm.nih.gov/geo/query/acc.cgi?acc=GSE224273> |
| LINCS L1000 | Subramanian et al.[27] | <https://pubmed.ncbi.nlm.nih.gov/29195078/> |
| **Experimental models: Organisms/strains** | | |
| Mouse: *CD4-^Cre+^/Prdm1^flox/flox^* | Salehi et al.[28] | N/A |
| Mouse: *Prdm1^flox/flox^* | Salehi et al.[28] | N/A |
| Mouse: LDLr^-/-^ | Jackson Laboratory | Strain #: 002207; RRID:IMSR_JAX:002207 |
| **Cell line** | | |
| Jurkat Clone E6­1 | ATCC® | Cat#TIB­152™ |
| **Software and algorithms** | | |
| Leica Q500MC | Leica Microsystems | N/A |
| Illumina BeadStudio v3.0 | Illumina, Inc. | N/A |
| lumi v2.38.0 | Du et al.[29] | <https://bioconductor.org/packages/release/bioc/html/lumi.html> |
| WGCNA v1.73 | Langfelder et al.[30] | <https://cran.r-project.org/web/packages/WGCNA/index.html> |
| bnlearn v4.5 | Scutari et al.[31] | <https://cran.r-project.org/web/packages/bnlearn/index.html> |
| limma v3.42.2 | Ritchie et al.[32] | <https://bioconductor.org/packages/release/bioc/html/limma.html> |
| clusterProfiler v3.12.0 | Yu et al.[33] | <https://guangchuangyu.github.io/software/clusterProfiler> |
| GOSemSim v2.20.0 | Yu et al.[34] | <https://github.com/YuLab-SMU/GOSemSim> |
| GENIE3 v1.6.0 | Huynh-Thu et al.[35] | <https://bioconductor.org/packages/release/bioc/html/GENIE3.html> |
| minet v3.42.0 | Meyer et al.[36] | <https://www.bioconductor.org/packages/release/bioc/html/minet.html> |
| iRegulon v1.3 | Janky et al.[37] | <http://iregulon.aertslab.org> |
| Cytoscape v3.8 | Shannon et al.[38] | <https://cytoscape.org> |
| PRESTO v1.1 | McArdle et al.[39] | <https://github.com/saramcardle/PRESTO> |
| Gephi v0.9.2 | Bastian et al.[40] | <https://gephi.org> |
| sra-tools v3.1.1 | National Center for Biotechnology Information (NCBI) | <https://github.com/ncbi/sra-tools> |
| Cell Ranger v8.0.0 | 10X Genomics, Inc. | <https://www.10xgenomics.com/support/software/cell-ranger/latest> |
| Seurat v5.1.0 | Hao et al.[41] | <https://satijalab.org/seurat> |
| DoubletFinder v2.0.4 | McGinnis et al.[42] | <https://github.com/chris-mcginnis-ucsf/DoubletFinder> |
| Harmony v1.2.0 | Korsunsky et al.[43] | <https://github.com/immunogenomics/harmony> |
| QuPath (v0.4.2) | [Bankheid](https://github.com/qupath/qupath) et al.[44] | <https://github.com/qupath/qupath> |
| GraphPad Prism 7 | GraphPad Software | https://www.graphpad.com |
| BD FACSDiva | BD Biosciences | <https://www.bdbiosciences.com/en-us/products/software/instrument-software/bd-facsdiva-software> |
| FlowJo 10.10.0™ | BD Biosciences | https://www.flowjo.com/solutions/flowjo |
