# Supplementary material for: Identification of a PRDM1-regulated T cell network to regulate atherosclerotic plaque inflammation
Source: Genome Med. 2025 Oct 2;17:109. doi: 10.1186/s13073-025-01541-6 (PMC12490039; doi:10.1186/s13073-025-01541-6)
Supplement: Supplementary file 5 — Additional file 5: Fig S1-S7 - Fig S1. Plaque stage classification. Representative image of the classification of the plaques stage in the aortic root: I - early progressive (pathologic intimal thickening, PIT); II - advanced with a thick fibrotic cap (TkF); III - advanced with a thin fibrotic cap (TnF). Scale bar = 500 µm. Fig S2. Additional results of unstable plaque WGCNA co-expression network. (a-b) Hierarchical clustering based on the eigengenes of unstable plaque WGCNA clusters (a) and stable plaque WGCNA clusters (b), shown as dendrograms. Fig S3. Comparison between stable and unstable WGCNA clusters. (a) For the top 10 stable co-expression clusters, the significance levels of the top-ranked overrepresented GO terms per cluster were visualized as a dot plot. (b) Venn diagrams showing gene overlap between selected stable and unstable clusters. (c) Heatmap shows Jaccard Index between all stable and unstable clusters. The number in square brackets indicates the size of the cluster. Four pairs of stable and unstable clusters highlighted are selected for the Venn Diagram visualization in (b). Significance level in (b-c) was evaluated by hypergeometric testing. Fig S4. Heatmaps of the unstable WGCNA clusters. Heatmaps showing the expression of genes in UP-32, UP-22, and UP-11 between unstable and stable plaques. For clarity sake, only core enrichment genes contributed to GSEA are shown. Fig S5. Relative cluster adjacency in stable plaques. Bar plot depicting the relative average adjacency of the UP-11 cluster, UP-22 cluster, and remaining clusters (“rest”) to the T cell-specific cluster (UP-32) based on the WGCNA adjacency matrix of the stable plaque network. The average adjacency between UP-32 and all other clusters was normalized to 1 and used as the reference. Fig S6. UP-32 gene co-expression cluster is enriched in plaque T cells (GSE224273). (a) UMAP plot shows carotid plaque cells from asymptomatic (n = 2) and symptomatic patients (n = 4) in GSE224273 dataset. [file 13073_2025_1541_MOESM5_ESM.docx]

**Additional file 5**


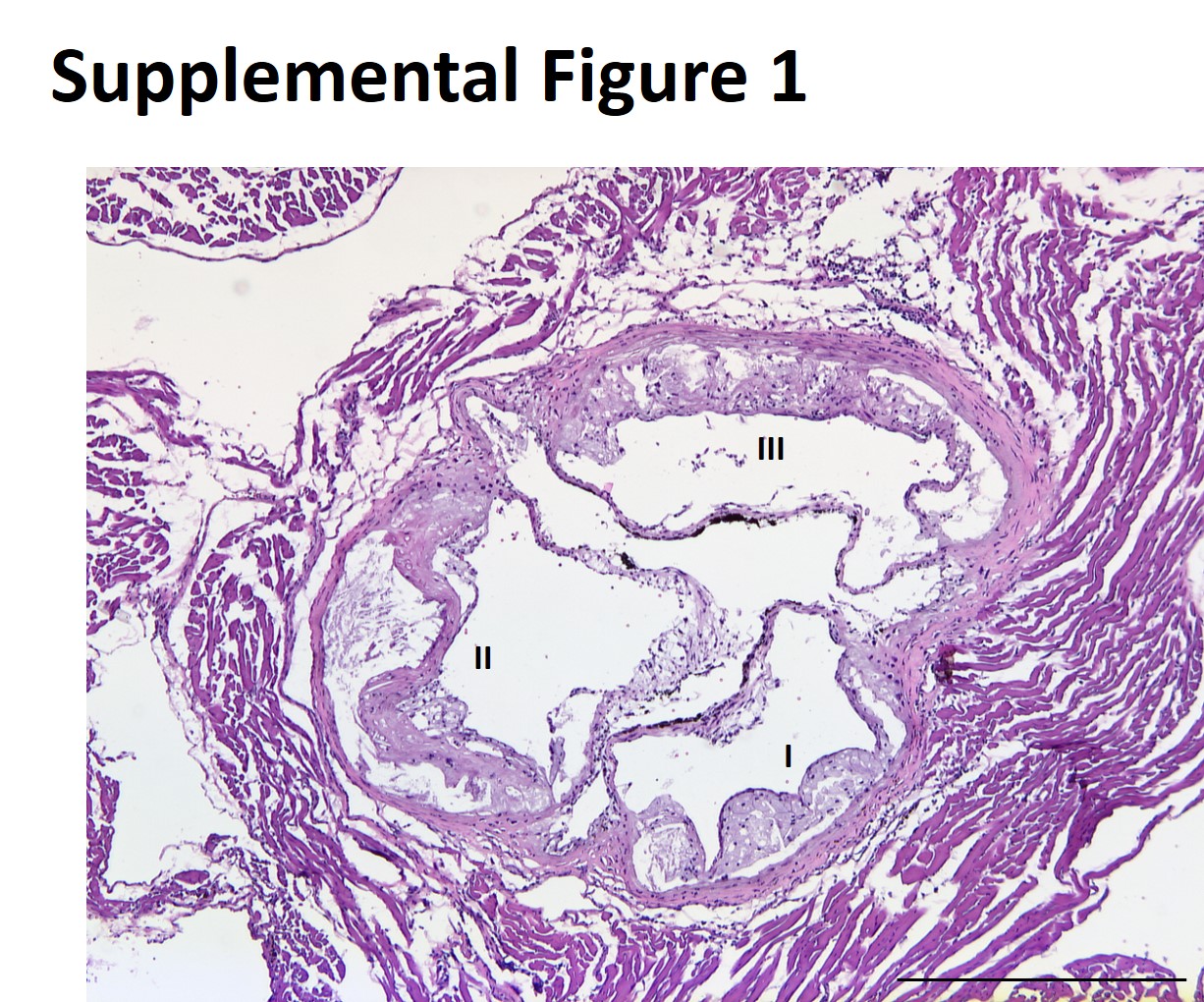


Fig S1. Plaque stage classification.

Representative image of the classification of the plaques stage in the aortic root: I - early progressive (pathologic intimal thickening, PIT); II - advanced with a thick fibrotic cap (TkF); III - advanced with a thin fibrotic cap (TnF). Scale bar = 500 µm.


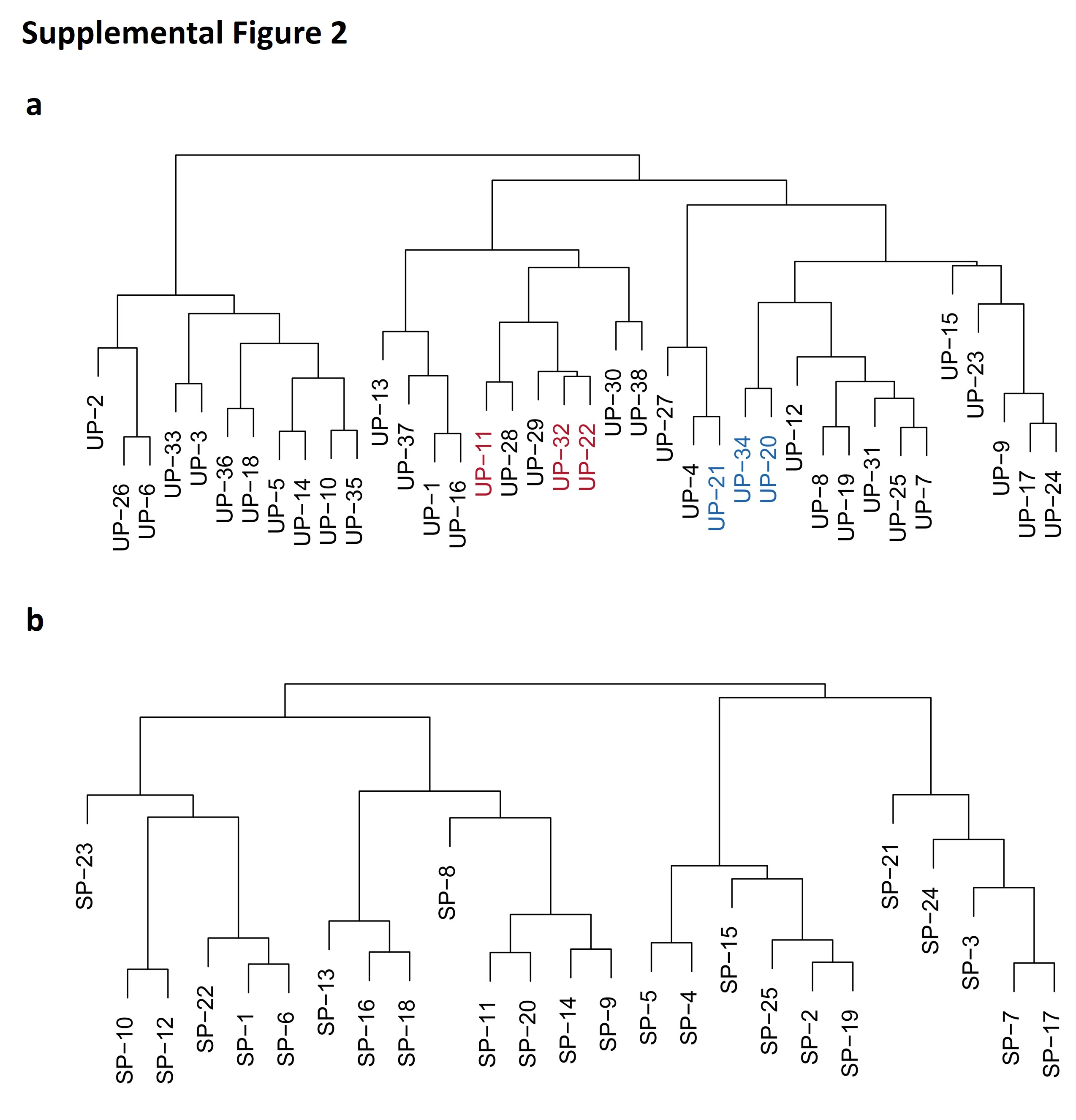


Fig S2. Additional results of unstable plaque WGCNA co-expression network

(a-b) Hierarchical clustering based on the eigengenes of unstable plaque WGCNA clusters (a) and stable plaque WGCNA clusters (b), shown as dendrograms.


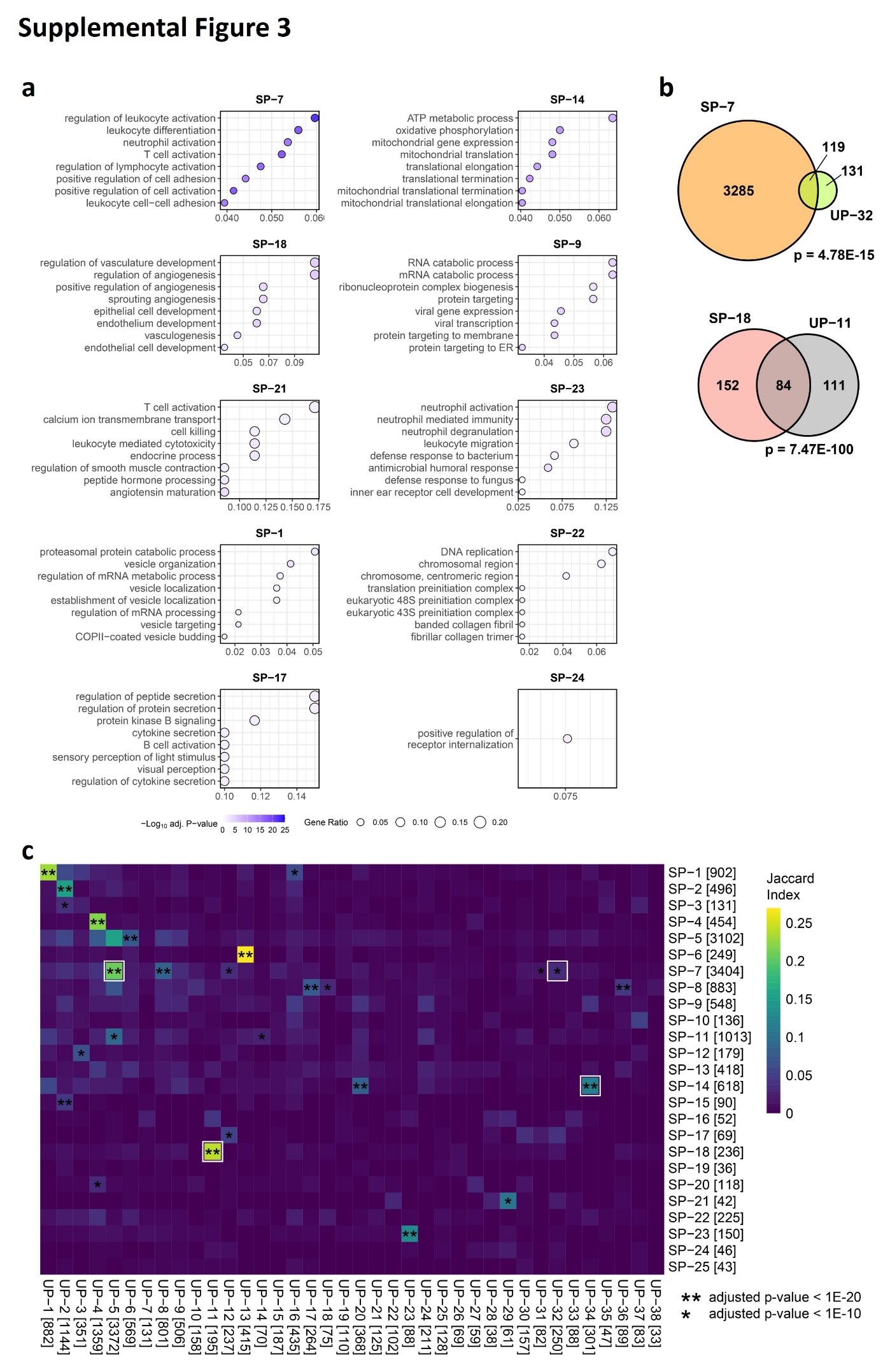


**Fig S3. Comparison between stable and unstable WGCNA clusters**

(a) For the top 10 stable co-expression clusters, the significance levels of the top-ranked overrepresented GO terms per cluster were visualized as a dot plot. (b) Venn diagrams showing gene overlap between selected stable and unstable clusters. (c) Heatmap shows Jaccard Index between all stable and unstable clusters. The number in square brackets indicates the size of the cluster. Four pairs of stable and unstable clusters highlighted are selected for the Venn Diagram visualization in (b). Significance level in (b-c) was evaluated by hypergeometric testing.


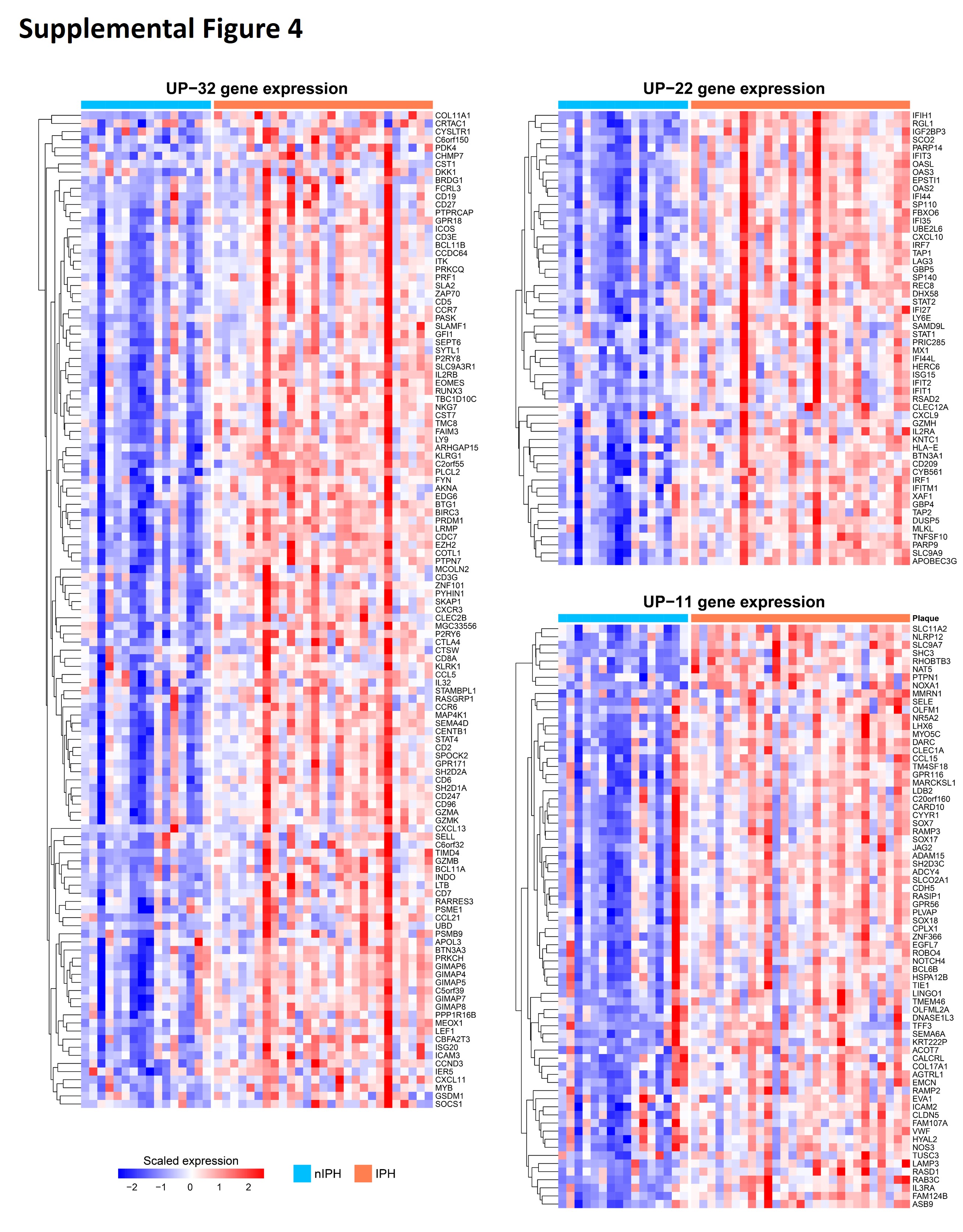


Fig S4. Heatmaps of the unstable WGCNA clusters

Heatmaps showing the expression of genes in UP-32, UP-22, and UP-11 between unstable and stable plaques. For clarity sake, only core enrichment genes contributed to GSEA are shown.


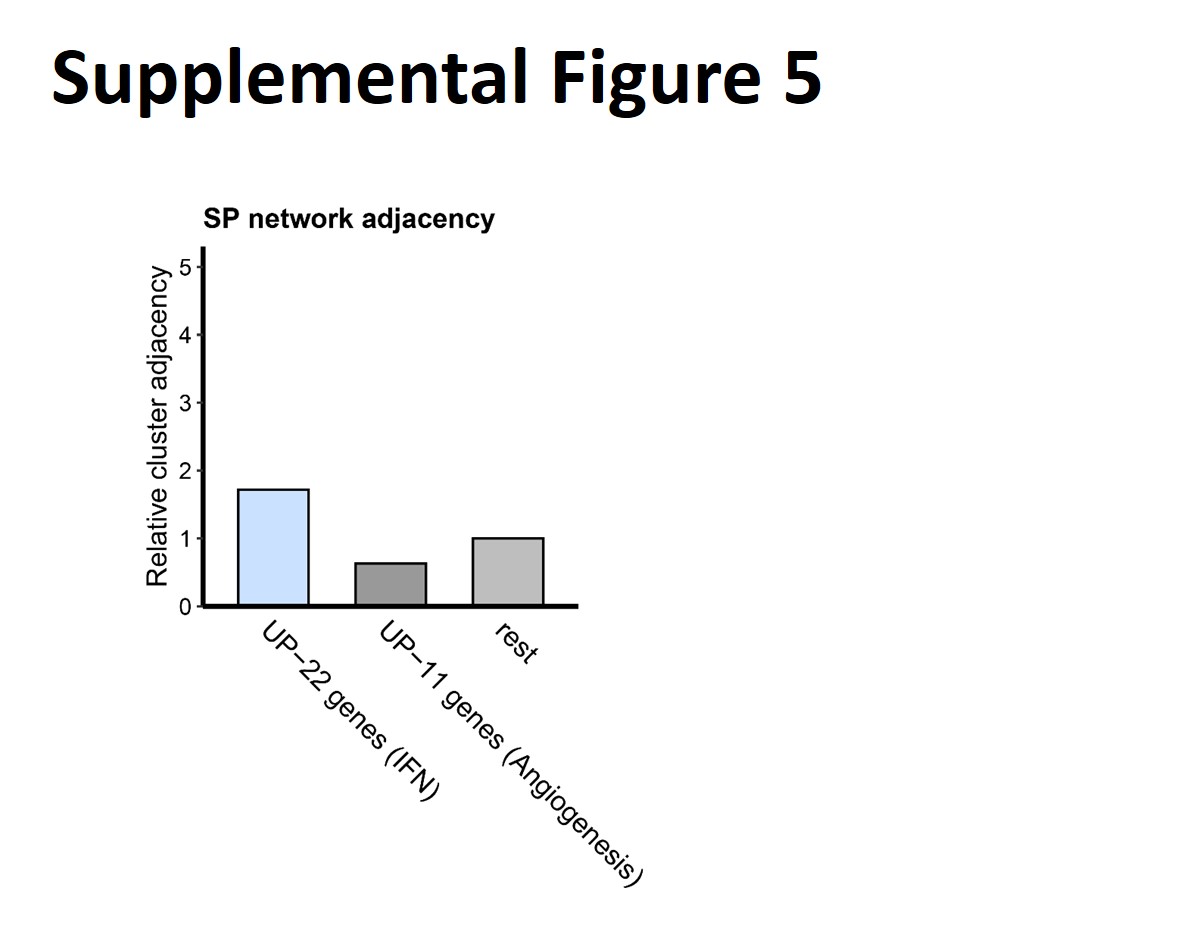


Fig S5. Relative cluster adjacency in stable plaques

Bar plot depicting the relative average adjacency of the UP-11 cluster, UP-22 cluster, and remaining clusters (“rest”) to the T cell-specific cluster (UP-32) based on the WGCNA adjacency matrix of the stable plaque network. The average adjacency between UP-32 and all other clusters was normalized to 1 and used as the reference.


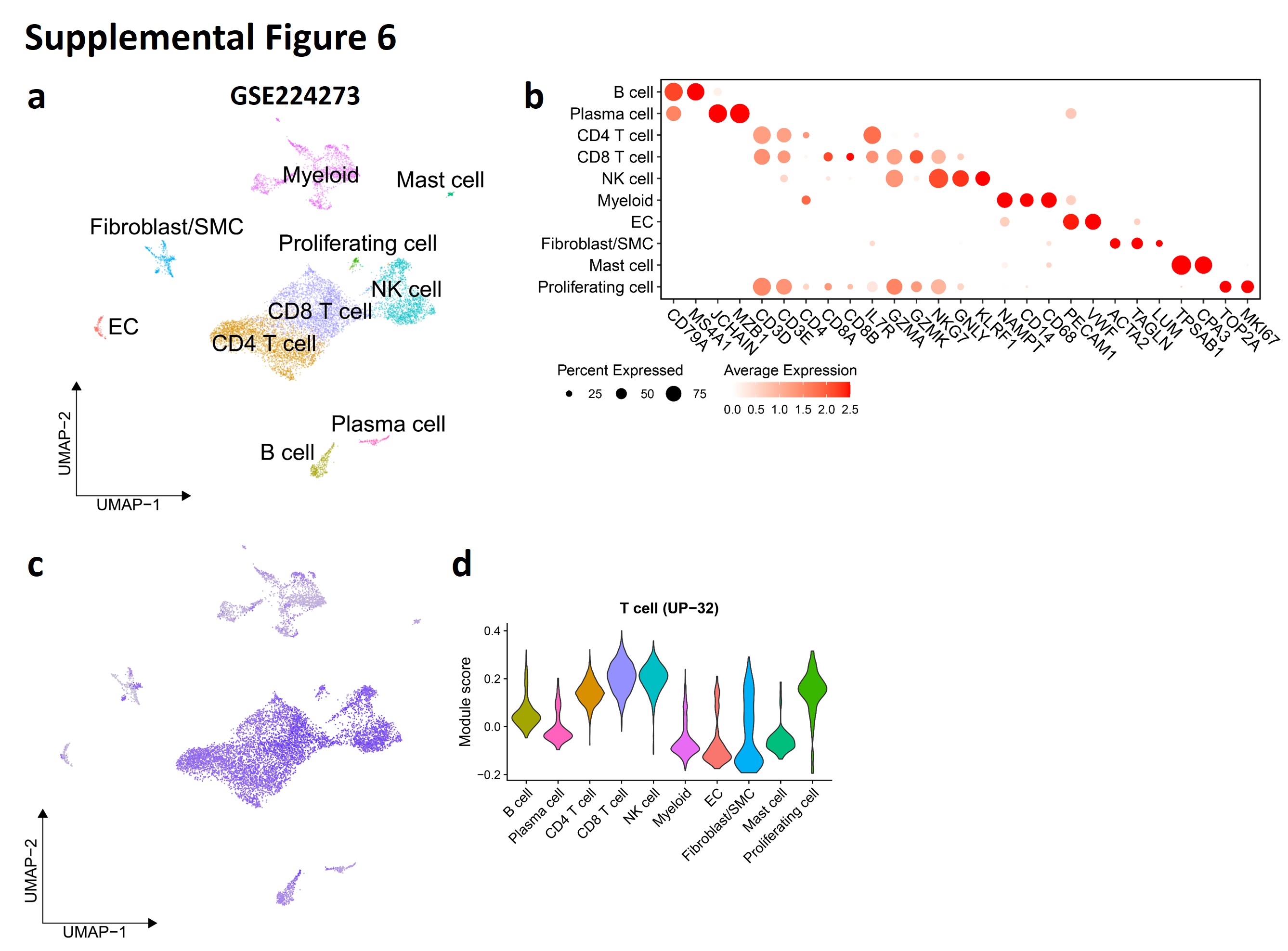


Fig S6. UP-32 gene co-expression cluster is enriched in plaque T cells (GSE224273)

(a) UMAP plot shows carotid plaque cells from asymptomatic (n = 2) and symptomatic patients (n = 4) in GSE224273 dataset. (b) Dot plot shows the markers of each cell type in (a). (c) UMAP plot shows the enrichment of the UP-32 T cell-specific cluster (UP-32; n = 250) in cells from GSE224273 dataset. (d) Violin plot shows the enrichment of the UP-32 T cell-specific cluster in plaque cells from GSE224273 dataset. EC – endothelial cell, NK – natural killer, SMC – smooth muscle cell.


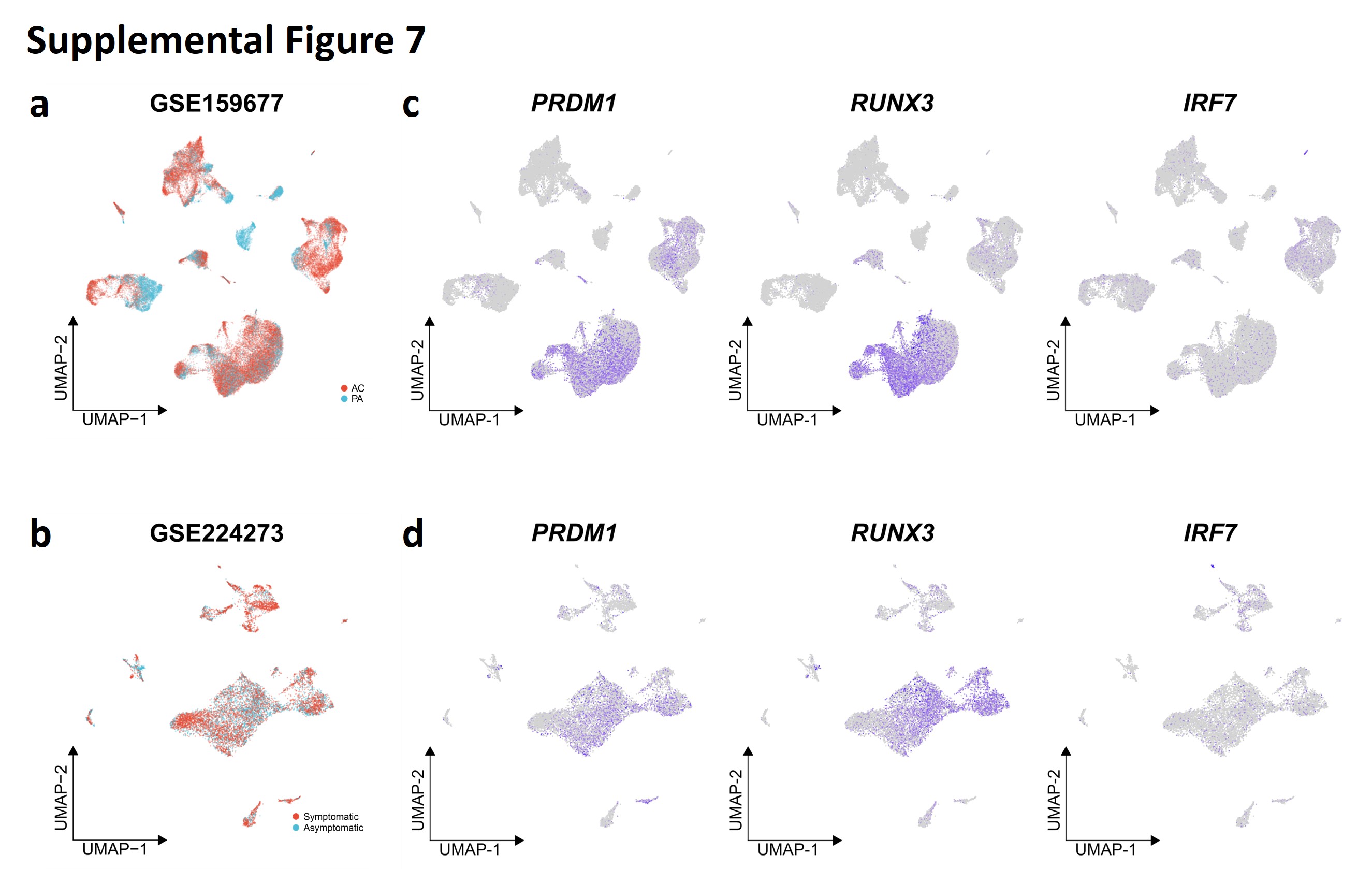


Fig S7. Expression of PRDM1, RUNX3 and IRF7 in plaque scRNA-seq datasets

(a) UMAP plots show cells from the atherosclerotic core (AC) and proximal adjacent (PA) tissues in GSE159677 dataset and (b) cells from the asymptomatic and symptomatic patients in GSE224273 dataset. (c-d) UMAP plots show the expression of PRDM1, RUNX3, and IRF7 in GSE159677 dataset (c) and GSE224273 dataset (d).
